# Temporal trends and mortality associated with difficult-to-treat resistance in bloodstream gram-negative bacilli: a retrospective cohort study in Southern Saudi Arabia

**DOI:** 10.3389/fcimb.2026.1919929

**Published:** 2026-08-31

**Authors:** Yahya Shabi, Ali M. Somily, Khalifa Binkhamis, Tarik Alazraqi, Safia Abdullah Mohammed, Sara Habbash, Norah Yahya Algathradi Asiri, Esmahan Tahtouh, Mona M Alshahrani, Ibrahim Tawhari, Soliuman Mohamed, Abdulah J. Alqahtani, Ihab M. Abdelrahim, Raghad Darwish, Abdulah OS Bawazeer

**Affiliations:** 1 Department of Microbiology and Clinical Parasitology, College of Medicine, King Khalid University, Abha, Saudi Arabia; 2 Department of Pathology, College of Medicine, King Saud University, Riyadh, Saudi Arabia; 3 Department of Internal Medicine, Aseer Central Hospital, Abha, Saudi Arabia; 4 Department of Medicine, College of Medicine, King Khalid University, Abha, Saudi Arabia; 5 Department of Haematology and Immunohaematology, Faculty of Medical Laboratory Sciences, University of Khartoum, Khartoum, Sudan; 6 College of Medicine, King Khalid University, Abha, Saudi Arabia; 7 Department of Microbiology, Aseer Central Hospital, Abha, Saudi Arabia

**Keywords:** antimicrobial resistance surveillance, bloodstream infection, carbapenem resistance, difficult-to-treat resistance, gram-negative bacteria, *Klebsiella pneumoniae*, retrospective cohort study, Saudi Arabia

## Abstract

**Introduction:**

Antimicrobial resistance among bloodstream gram-negative bacilli is a major driver of infectious-disease mortality, yet broad resistance labels can obscure clinically important heterogeneity. We aimed to characterise long-term organism and resistance trends, including difficult-to-treat resistance (DTR), among bloodstream gram-negative bacilli and to estimate their association with 30-day in-hospital mortality.

**Methods:**

We conducted a retrospective cohort study of hospitalized patients aged 12 years or older with blood cultures yielding Enterobacterales or non-fermenting gram-negative bacilli (GNB) at a 500-bed tertiary hospital in Southern Saudi Arabia from 1 January 2013 to 27 August 2024. Duplicates within 14 days were excluded; trends were analyzed with segmented binomial regression and mortality with modified Poisson regression adjusted for age, sex, intensive-care status, infection source, healthcare-associated infection, COVID-19 era, calendar year and Elixhauser comorbidity score.

**Results:**

Among 2027 isolate-episodes from 1695 patients, 1487 (73.4%) were Enterobacterales and 540 (26.6%) non-fermenting GNB. The organism mix was broadly stable, but *Klebsiella pneumoniae* was dominant (37.6%) and increased (average annual percentage change (AAPC) +4.8%). Fluoroquinolone and extended-spectrum cephalosporin resistance declined among Enterobacterales, whereas carbapenem resistance (46.9%) and DTR (36.0%) rose, most sharply among Enterobacterales. Thirty-day in-hospital mortality was 36.5%. DTR was independently associated with higher mortality among Enterobacterales (adjusted risk ratio 1.35; 95% CI 1.15 to 1.59) and non-fermenting GNB (1.31; 1.09 to 1.57), with a graded increase as active first-line agents were lost.

**Discussion:**

In this surveillance cohort, escalating carbapenem resistance and DTR, rather than a shifting organism mix, were associated with worse outcomes, supporting surveillance based on treatment-option phenotypes and prioritization of stewardship and infection-prevention efforts against high-risk GNB.

## 1 Introduction

Antimicrobial resistance (AMR) is a major contributor to infectious-disease mortality, and several leading AMR pathogen-drug combinations involve bloodstream-relevant gram-negative organisms, including third-generation cephalosporin-resistant *Escherichia coli* and *Klebsiella pneumoniae*, carbapenem-resistant *K. pneumoniae* and carbapenem-resistant *Acinetobacter baumannii* (Antimicrobial Resistance Collaborators, 2022). The 2024 World Health Organization bacterial priority pathogens list and Global Antimicrobial Resistance and Use Surveillance System (GLASS) reports similarly highlight last-resort antibiotic resistance in gram-negative bacteria as a central research and public-health priority (World Health Organization, 2022, 2024).

For hospitals, bloodstream isolates are a useful surveillance signal because they are clinically consequential, less prone to colonization-only ambiguity than many non-sterile specimens, and directly linked to mortality. However, surveillance based only on broad resistance labels can miss clinically relevant heterogeneity. Difficult-to-treat resistance (DTR), defined by non-susceptibility to all routinely used first-line agents, was proposed to describe a phenotype closer to bedside therapeutic constraint than multidrug resistance alone (Kadri et al., 2018).

International surveillance and multicentre cohort studies show why bloodstream gram-negative resistance should be interpreted by organism and treatment option phenotype. Global estimates identify resistant Enterobacterales and non-fermenting gram-negative bacilli as major contributors to AMR burden, while DTR bacteraemia has been associated with excess mortality in large hospital cohorts (Antimicrobial Resistance Collaborators, 2022; Kadri et al., 2018). These findings support evaluating DTR and related resistance categories as clinically interpretable markers of limited empiric and definitive treatment options.

Regional and hospital-level trend data remain necessary because organism distributions, antimicrobial pressure, laboratory methods and infection-prevention practices vary substantially across settings. Recent Saudi studies have described carbapenemase-producing and carbapenem-resistant *K. pneumoniae*, resistance trends among non-fermenting gram-negative bacilli, and clinical outcomes of *K. pneumoniae* bacteraemia, while Southern Saudi surveillance work has reported rising carbapenem and DTR phenotypes in *Klebsiella* species and clinically relevant resistance in Morganellaceae (Alghoribi et al., 2020; Alkhelaiwi et al., 2026; Hafiz et al., 2023; Shabi et al., 2025, 2026a, b; Somily et al., 2021). These data support local analyses that link organism-resistance phenotypes with patient outcomes rather than extrapolating from global estimates alone.

Long-term, decade-scale single-center data linking treatment-limiting resistance phenotypes—including DTR and carbapenem resistance—to patient-level mortality remain scarce for Southern Saudi Arabia and the broader region, and most regional reports to date are cross-sectional or organism-specific and do not link resistance phenotype to individual-patient outcomes. The aim of this study was to characterize long-term organism and resistance trends among bloodstream gram-negative bacilli at a tertiary hospital in Southern Saudi Arabia and to estimate the association between antimicrobial resistance phenotypes, including DTR, and 30-day in-hospital mortality. The primary expectation was that resistance phenotypes reflecting fewer active first-line options would be associated with higher mortality after adjustment for measured patient and admission-level factors.

## 2 Materials and methods

### 2.1 Study design, setting and participants

We conducted a retrospective cohort study at Aseer Central Hospital, a 500-bed tertiary-care facility serving the Aseer region of Southern Saudi Arabia. The study followed STROBE guidance for observational studies and was designed as a culture-based surveillance analysis rather than a clinically adjudicated sepsis cohort (von Elm et al., 2007). The source population comprised hospitalized patients aged 12 years or older with blood cultures yielding GNB between 1 January 2013 and 27 August 2024. Emergency-department cultures were included only if the encounter resulted in hospital admission; outpatient cultures and patients younger than 12 years were excluded.

For isolate-level trend analyses, same-species isolates from the same patient within a 14-day window were considered duplicate isolate-episodes and only the first was retained. For baseline and mortality analyses, the unit of analysis was the first bloodstream GNB culture per admission. This avoided counting the same admission repeatedly and reduced bias from follow-up cultures that may reflect persistent bacteraemia, treatment failure or repeat testing.

### 2.2 Laboratory methods and resistance definitions

Organisms were grouped as Enterobacterales or non-fermenting GNB using taxonomic assignment (Adeolu et al., 2016; Parte et al., 2020) and glucose fermentation status. Identification and antimicrobial susceptibility testing (AST) was performed with the VITEK 2 system (bioMérieux, Marcy-l’Étoile, France) using gram-negative identification and AST cards. MALDI-TOF MS was not available during the study period. Minimum inhibitory concentrations were interpreted using the Clinical and Laboratory Standards Institute (CLSI) breakpoints in use at the time of testing; raw MIC values were not available for retrospective reinterpretation under a single breakpoint standard (Clinical and Laboratory Standards Institute, 2026). Intermediate and resistant results were analyzed as non-susceptible.

#### 2.2.1 Laboratory quality assurance and quality control

Identification and antimicrobial susceptibility testing were performed using the VITEK 2 system according to the manufacturer’s instructions and CLSI M100 recommendations. Internal quality control was performed according to the microbiology laboratory standard operating procedures using reference strains including *Escherichia coli* ATCC 25922, *Pseudomonas aeruginosa* ATCC 27853, *Klebsiella pneumoniae* ATCC 700603, and *Enterococcus faecalis* ATCC 29212. QC results were accepted only when results fell within the manufacturer- and CLSI-specified ranges. Failed QC results triggered repeat testing and review of inoculum preparation, card lot, instrument status, and maintenance records before patient results were released. Neither phenotypic carbapenemase confirmation (e.g., the modified carbapenem inactivation method or Carba NP test) nor molecular carbapenemase detection was performed; carbapenem resistance and difficult-to-treat resistance were classified solely from VITEK 2 antimicrobial susceptibility results, interpreted using the CLSI breakpoints in use at the time of testing and the reference phenotype definitions applied in this study (Kadri et al., 2018).

Fluoroquinolone resistance (FQR) was defined as non-susceptibility to ciprofloxacin or levofloxacin. Extended-spectrum cephalosporin resistance (ESC-R) was restricted to Enterobacterales and defined as non-susceptibility to ceftriaxone, cefotaxime, ceftazidime or cefepime. Carbapenem resistance was defined as non-susceptibility to any applicable carbapenem: imipenem, meropenem or ertapenem for non-Proteeae Enterobacterales; meropenem or ertapenem for Proteeae; and imipenem or meropenem for *P. aeruginosa* and *A. baumannii*. DTR was defined for Enterobacterales, *P. aeruginosa* and *A. baumannii* using criteria adapted from Kadri et al. (2018), requiring non-susceptibility across organism-appropriate first-line categories. The active-agent framework counted carbapenems, extended-spectrum cephalosporins, fluoroquinolones, piperacillin-tazobactam when tested, and aztreonam for non-*Acinetobacter* species or ampicillin-sulbactam for *A. baumannii* when tested. *Stenotrophomonas maltophilia* and *Burkholderia cepacia* complex were excluded from the DTR definition and from resistance-hierarchy and active-agent analyses because the standard first-line category framework was not applicable.

### 2.3 Outcomes

The co-primary outcomes were the annual proportion of Enterobacterales among bloodstream GNB isolates and 30-day in-hospital mortality by organism-resistance composite category. Mortality was defined as death during the index admission within 30 days after blood culture collection. Secondary outcomes included species-level trends, temporal changes in FQR, ESC-R, carbapenem resistance and DTR, mortality associations across a five-level resistance hierarchy, and a dose-response analysis based on the number of active first-line antimicrobial categories.

### 2.4 Statistical analysis

Categorical variables were summarized as frequencies and percentages; continuous variables were summarized as medians with interquartile ranges. Temporal trends were modelled within the segmented (joinpoint) regression framework of Kim et al. (2000, 2023), implemented here as a binomial regression with a log link allowing up to two joinpoints. Annual percentage change and average annual percentage change (AAPC) were estimated with 95% confidence intervals (CIs) using delta-method variance estimation (Clegg et al., 2009). Subgroup trend analyses by sex and intensive-care-unit (ICU) setting were performed only when prespecified event-count and denominator thresholds were met.

Mortality associations were estimated using modified Poisson regression with robust standard errors clustered by patient (Zou, 2004). Models were adjusted for age, sex, ICU status at culture, potential bloodstream infection source, healthcare-associated infection, COVID-19 era, calendar year and the Elixhauser Comorbidity Index using van Walraven weighting (Elixhauser et al., 1998; van Walraven et al., 2009). A potential source was defined as a concordant non-blood culture of the same species within 7 days before or after the blood culture. Healthcare-associated infection was defined as culture collection more than 2 days after admission, adapted from CDC/NHSN timing rules (Centers for Disease Control and Prevention, 2026). Calendar year and the COVID-19-era indicator were retained as separate covariates because they capture complementary temporal signals: calendar year models the smooth secular trend in organism distribution, resistance and case-mix across the 12-year study period, whereas the COVID-19-era indicator (2020–2024) captures a discrete, period-specific shift in case-mix, admission pathways and infection-control practice associated with the pandemic. Because calendar year enters the model as a continuous secular trend whereas the COVID-19-era indicator marks a single 2020 threshold, the two terms capture distinct temporal information and were retained together; neither was strongly associated with mortality after adjustment (see Results), indicating that collinearity did not destabilize the estimates. Continuous covariates were winsorized, standardized and assessed for non-linearity using restricted cubic splines and fractional polynomials. Analyses used Stata 19.5 (StataCorp LLC) and Python 3.12. Detailed organism-antibiotic exclusion rules, resistance-phenotype and DTR derivations, the segmented (joinpoint) model specification and selection, and all supplementary analyses are provided in the eMethods and **Supplementary Materials** (**Supplementary Tables 1**-**14**; **Supplementary Figures 1**-**9**).

Analyses were conducted using complete case data for the variables required by each model; susceptibility data were not imputed. The proportion of isolates with an interpretable susceptibility result was quantified for each antimicrobial category contributing to the resistance-phenotype and difficult-to-treat resistance (DTR) definitions; among the 2027 isolate-episodes, testing coverage for the principal first-line agents ranged from approximately 80% to 93% (meropenem 1843/2027 [90.9%], imipenem 1788/2027 [88.2%], ciprofloxacin 1880/2027 [92.7%], ceftazidime 1635/2027 [80.7%] and piperacillin–tazobactam 1626/2027 [80.2%]) and was lower for less routinely tested agents (e.g., aztreonam 736/2027 [36.3%]; full per-agent denominators in **Supplementary Table 4**). At the phenotype level, isolates were evaluable for carbapenem resistance in 1890/2027 (93.2%) and for DTR in 1691/2027 (83.4%). Consistent with the complete-case approach, isolates lacking a susceptibility result for a given category were excluded from classification within that category rather than imputed. Sensitivity analyses included overall in-hospital mortality, first admission per patient, exclusion of ICU from covariables, exclusion of potential source from covariables, and last rather than first culture per admission.

## 3 Results

### 3.1 Study population

**Figure 1:**
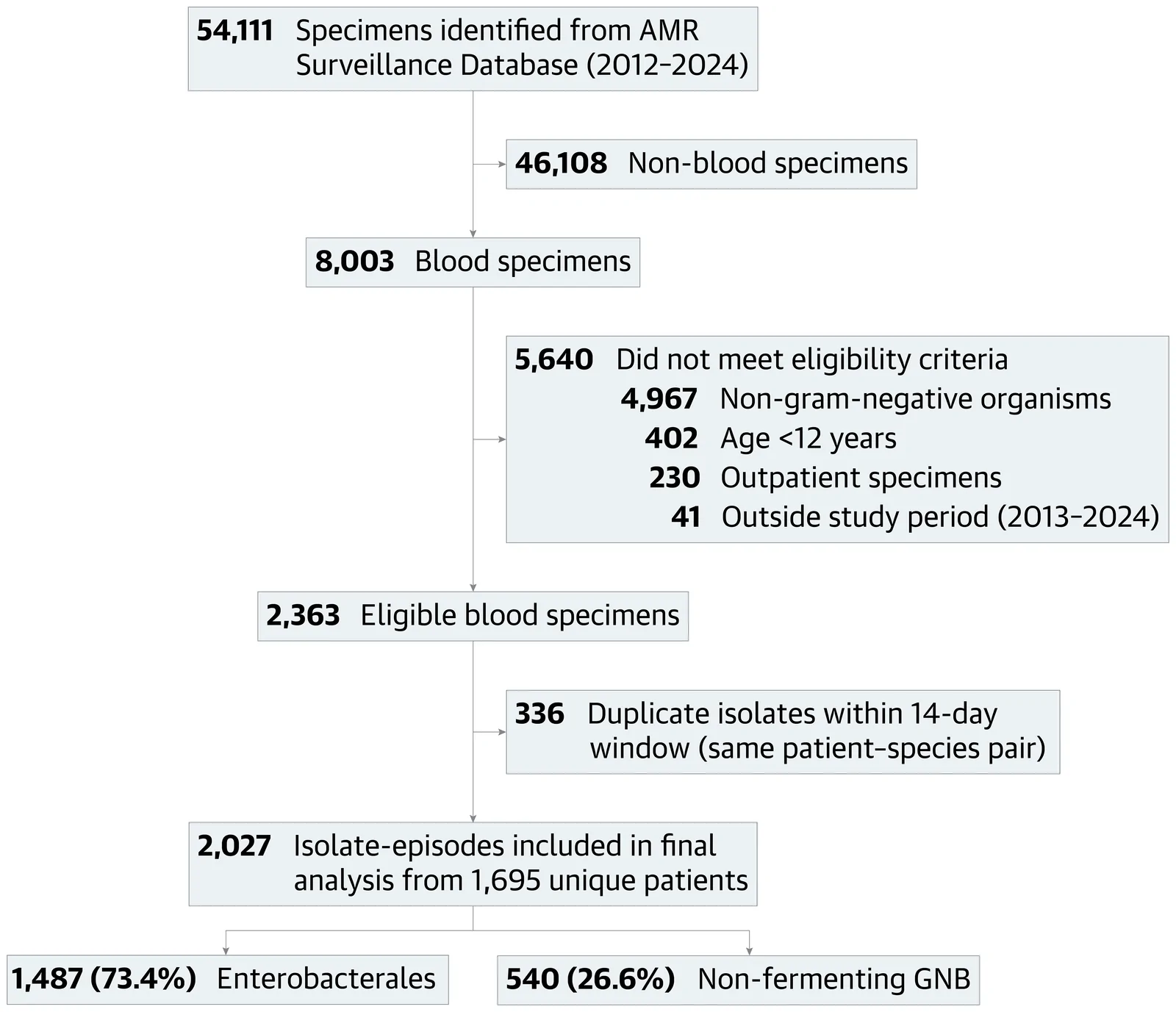
Study flow diagram.

The institutional AMR surveillance database contained 54,111 isolates from 2012 to 2024. After applying study-period, specimen, age and admission criteria, 2363 eligible blood GNB isolates remained. Exclusion of 336 duplicate same-patient same-species isolate-episodes left 2027 isolate-episodes from 1695 unique patients (**Figure 1**). Enterobacterales accounted for 1487/2027 (73.4%) and non-fermenting GNB for 540/2027 (26.6%).

The admission-level cohort comprised 1728 first bloodstream GNB cultures. Median age was 60 years (IQR 39-74), 1133/1728 (65.6%) were in men, 878/1728 (50.8%) of cultures were obtained in the ICU, and 1179/1728 (68.2%) were healthcare-associated (**Table 1**). The median hospital length of stay was 25 days (IQR 12-48). A potential bloodstream infection source was present in 718/1728 (41.6%), most commonly respiratory or urinary among source-identified episodes.

**Table 1:** Baseline characteristics of the admission-level bloodstream gram-negative bacilli cohort (N = 1728).

Characteristic	Total N = 1728	Enterobacterales n=1254	Non-fermenting GNB n=474
Age, years, median (IQR)	60 (39-74)	62 (40-75)	54 (34-72)
Men	1133 (65.6)	813 (64.8)	320 (67.5)
ICU at culture	878 (50.8)	630 (50.2)	248 (52.3)
Healthcare-associated (>2 days)	1179 (68.2)	823 (65.6)	356 (75.1)
COVID-19 era (2020-2024)	940 (54.4)	724 (57.7)	216 (45.6)
Length of stay, days, median (IQR)	25 (12-48)	24 (12-47)	28 (13-51)
Days from admission to culture, median (IQR)	10 (1-25)	10 (1-27)	10 (3-22)
Potential BSI source identified	718 (41.6)	560 (44.7)	158 (33.3)
Peripheral blood culture	1646 (95.3)	1208 (96.3)	438 (92.4)
Central-line culture	82 (4.7)	46 (3.7)	36 (7.6)
Hypertension	693 (40.1)	522 (41.6)	171 (36.1)
Diabetes mellitus	304 (17.6)	242 (19.3)	62 (13.1)
Renal failure	212 (12.3)	157 (12.5)	55 (11.6)
Malignancy	189 (10.9)	152 (12.1)	37 (7.8)
Elixhauser Comorbidity Index, median (IQR)	0 (0-4)	0 (0-4)	0 (0-4)

Values are n (%) unless otherwise stated. Potential BSI source was defined as a concordant non-blood culture of the same species within +/-7 days of blood culture. ICU, intensive care unit; IQR, interquartile range.

### 3.2 Organism and resistance trends

**Figure 2:**
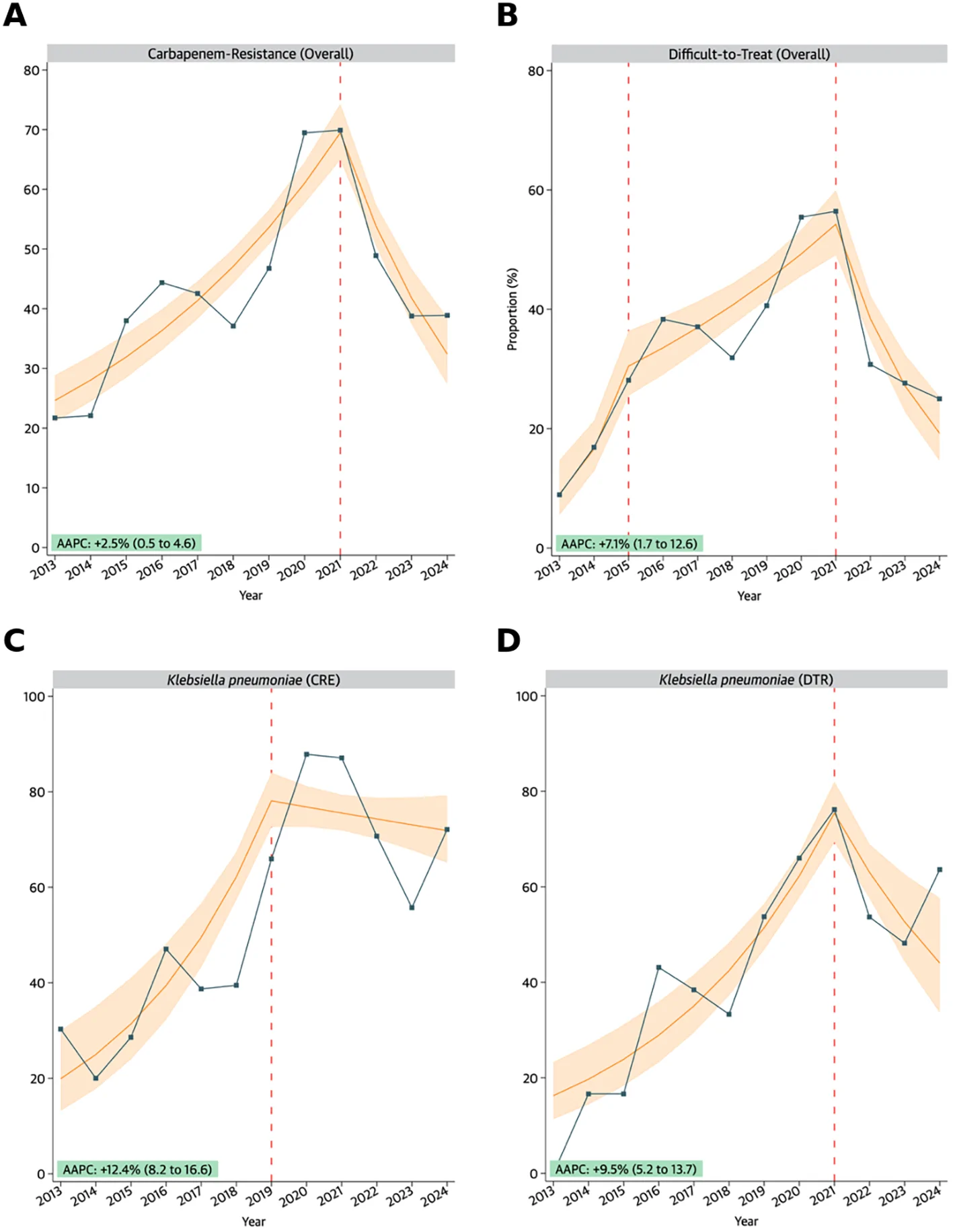
Temporal trends in carbapenem resistance and difficult-to-treat resistance (DTR) among bloodstream gram-negative bacilli, 2013–2024. Observed annual proportions (connected markers) with segmented (joinpoint) binomial regression fits (line) and 95% confidence bands; the average annual percentage change (AAPC) with 95% CI is annotated in each panel and red dashed lines mark estimated joinpoints. **(A)** Carbapenem resistance, all gram-negative bacilli; **(B)** DTR, all gram-negative bacilli; **(C)** carbapenem-resistant *Klebsiella pneumoniae*; **(D)** DTR *Klebsiella pneumoniae*.

The overall proportion of Enterobacterales among bloodstream GNB remained stable (AAPC + 0.5%; 95% CI -0.3 to 1.3), as did the proportion of non-fermenting GNB (AAPC -1.4%; 95% CI -4.3 to 1.4). *K. pneumoniae* was the most common species (763/2027, 37.6%) and increased over time (AAPC + 4.8%; 95% CI 2.3 to 7.3), with a breakpoint at 2021. *E. coli* was stable (315/2027, 15.5%; AAPC + 2.6%; 95% CI -0.4 to 5.6). Among non-fermenters, *A. baumannii* (277/2027, 13.7%) and *P. aeruginosa* (176/2027, 8.7%) declined (**Figure 2**; **Table 2**; **Supplementary Tables 6**, **7**; **Supplementary Figure 2**-**4**).

**Table 2:** Selected organism and resistance trends among bloodstream gram-negative bacilli, 2013-2024.

Outcome	Evaluable N	n (%)	Breakpoint(s)	AAPC % (95% CI)	Direction
Enterobacterales proportion	2027	1487 (73.4)	Linear	+0.5 (-0.3 to 1.3)	Stable
Non-fermenting GNB proportion	2027	540 (26.6)	Linear	-1.4 (-4.3 to 1.4)	Stable
*K. pneumoniae*	2027	763 (37.6)	2021	+4.8 (2.3 to 7.3)	Increasing
*E. coli*	2027	315 (15.5)	Linear	+2.6 (-0.4 to 5.6)	Stable
*A. baumannii*	2027	277 (13.7)	Linear	-4.0 (-7.0 to -1.1)	Decreasing
*P. aeruginosa*	2027	176 (8.7)	Linear	-6.4 (-10.2 to -2.6)	Decreasing
FQR, Enterobacterales	1437	928 (64.6)	2019, 2021	-3.7 (-5.3 to -2.1)	Decreasing
ESC-R, Enterobacterales	1292	892 (69.0)	2021	-3.5 (-4.9 to -2.0)	Decreasing
Carbapenem resistance, all isolates	1890	887 (46.9)	2021	+2.5 (0.5 to 4.6)	Increasing
Carbapenem resistance, Enterobacterales	1447	595 (41.1)	2021	+7.0 (4.0 to 10.1)	Increasing
Carbapenem-resistant *K. pneumoniae*	743	493 (66.4)	2019	+12.4 (8.2 to 16.6)	Increasing
DTR, all evaluable isolates	1691	608 (36.0)	2015, 2021	+7.1 (1.7 to 12.6)	Increasing
DTR, Enterobacterales	1258	403 (32.0)	2016, 2021	+23.8 (10.9 to 36.8)	Increasing
DTR *K. pneumoniae*	680	368 (54.1)	2021	+9.5 (5.2 to 13.7)	Increasing
DTR *A. baumannii*	265	178 (67.2)	2020	-11.3 (-16.0 to -6.5)	Decreasing

AAPC, average annual percentage change, was estimated from segmented binomial regression with log link. Direction is based on whether the 95% CI excludes zero. FQR, fluoroquinolone resistance; ESC-R, extended-spectrum cephalosporin resistance; DTR, difficult-to-treat resistance.

Resistance trends were not uniform. Among Enterobacterales, FQR declined (AAPC -3.7%; 95% CI -5.3 to -2.1) and ESC-R declined (AAPC -3.5%; 95% CI -4.9 to -2.0). In contrast, carbapenem resistance was present in 887/1890 evaluable isolates (46.9%) and increased overall (AAPC + 2.5%; 95% CI 0.5 to 4.6). Carbapenem resistance increased more clearly among Enterobacterales (595/1447, 41.1%; AAPC + 7.0%; 95% CI 4.0 to 10.1), driven by *K. pneumoniae* (493/743, 66.4%; AAPC + 12.4%; 95% CI 8.2 to 16.6).

DTR was present in 608/1691 DTR-evaluable isolates (36.0%) and increased over the study period (AAPC + 7.1%; 95% CI 1.7 to 12.6). DTR increased sharply among Enterobacterales (403/1258, 32.0%; AAPC + 23.8%; 95% CI 10.9 to 36.8) and among *K. pneumoniae* (368/680, 54.1%; AAPC + 9.5%; 95% CI 5.2 to 13.7), whereas DTR *A. baumannii* declined (178/265, 67.2%; AAPC -11.3%; 95% CI -16.0 to -6.5) (**Table 2**).

### 3.3 Mortality

**Figure 3:**
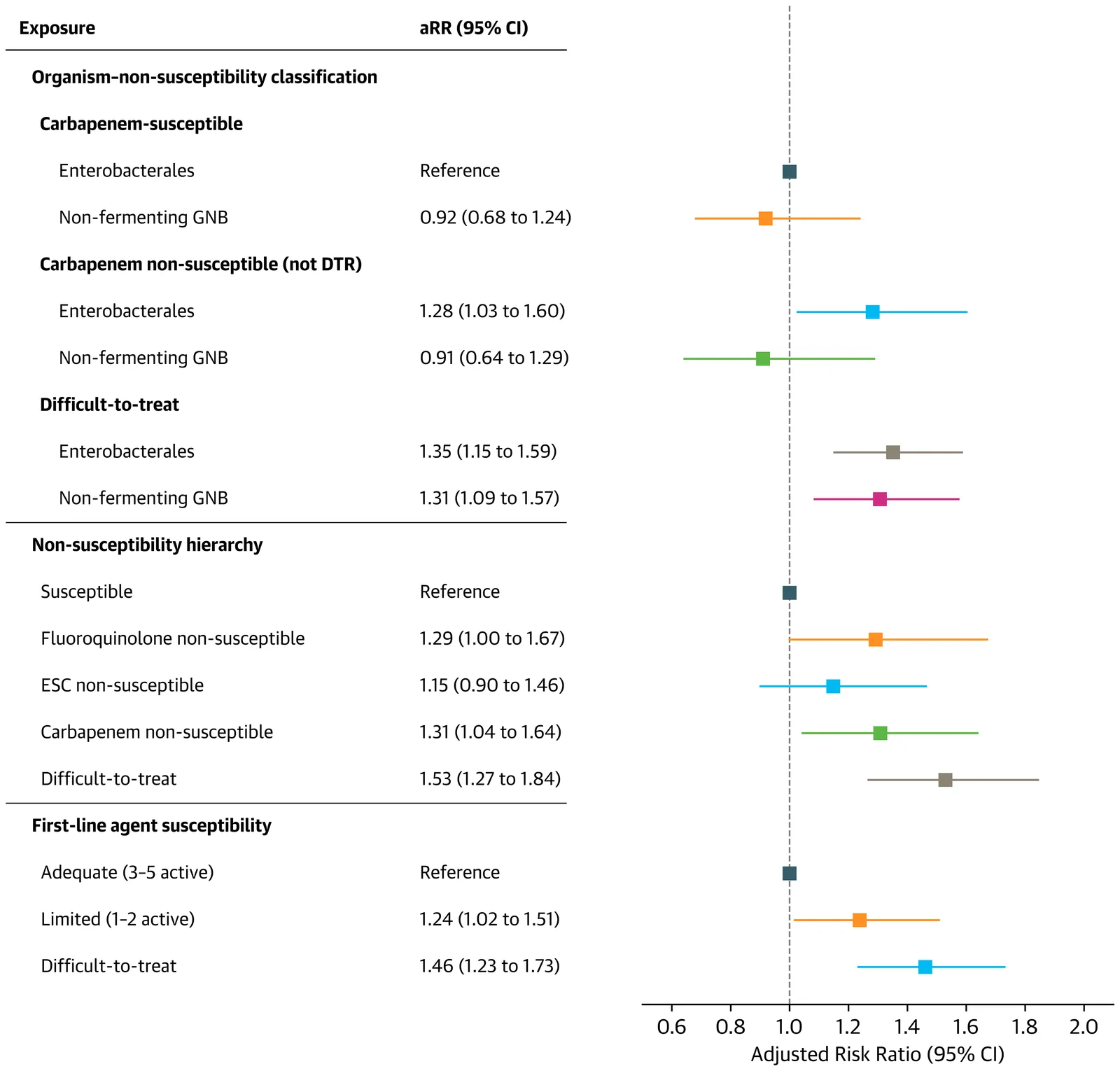
Adjusted risk ratios for 30-day in-hospital mortality across three resistance exposure frameworks among bloodstream gram-negative bacilli, 2013 to 2024. Squares represent adjusted risk ratios (aRR) and horizontal lines the corresponding 95% confidence intervals (CI), estimated using modified Poisson regression with robust standard errors clustered by patient and adjusted for age, sex, intensive care unit status at culture, potential bloodstream infection source, healthcare-associated infection, COVID-19 era, calendar year and the Elixhauser Comorbidity Index. The three frameworks are: the organism and non-susceptibility classification (N = 1573; reference, carbapenem-susceptible Enterobacterales); the non-susceptibility hierarchy (N = 1663; reference, fully susceptible isolates); and first-line agent susceptibility, defined by the number of active first-line antimicrobial categories (N = 1443; reference, adequate, 3 to 5 active categories). Reference categories are plotted at an aRR of 1.00. The vertical dashed line marks the null value of 1.00, and the x-axis shows the adjusted risk ratio on a scale from 0.6 to 2.0. aRR, adjusted risk ratio; CI, confidence interval; DTR, difficult-to-treat resistance; ESC, extended-spectrum cephalosporin; GNB, gram-negative bacilli.

Overall 30-day in-hospital mortality was 630/1728 (36.5%). The COVID-19 era (2020–2024) was not independently associated with 30-day in-hospital mortality in the fully adjusted models (adjusted risk ratio 1.12, 95% CI 0.91 to 1.38 in the composite model; P = 0.275), although crude mortality was modestly higher during this period (crude risk ratio 1.19, 95% CI 1.05 to 1.36; **Supplementary Table 9**). In the fully adjusted organism-resistance composite model (N = 1573), DTR was associated with higher mortality in both organism groups. Compared with carbapenem-susceptible Enterobacterales, DTR Enterobacterales had an adjusted risk ratio (aRR) of 1.35 (95% CI 1.15 to 1.59) and DTR non-fermenting GNB had an aRR of 1.31 (95% CI 1.09 to 1.57). Carbapenem-resistant non-DTR Enterobacterales were also associated with higher mortality (aRR 1.28; 95% CI 1.03 to 1.60) (**Table 3**; **Figure 3**; **Supplementary Table 9**; **Supplementary Figure 6**).

**Table 3:** Antimicrobial resistance and 30-day in-hospital mortality among bloodstream gram-negative bacilli.

Exposure	N	Died n (%)	Adjusted RR (95% CI)	P value
Organism-resistance composite (N = 1573)				
Carbapenem-susceptible Enterobacterales (reference)	727	192 (26.4)	1.00	–
Carbapenem-resistant Enterobacterales, non-DTR	127	58 (45.7)	1.28 (1.03 to 1.60)	0.028
DTR Enterobacterales	334	189 (56.6)	1.35 (1.15 to 1.59)	<0.001
Carbapenem-susceptible non-fermenting GNB	136	32 (23.5)	0.92 (0.68 to 1.24)	0.577
Carbapenem-resistant non-fermenting GNB, non-DTR	67	21 (31.3)	0.91 (0.64 to 1.29)	0.593
DTR non-fermenting GNB	182	89 (48.9)	1.31 (1.09 to 1.57)	0.005
Resistance hierarchy (N = 1663)				
Non-resistant (reference)	460	103 (22.4)	1.00	–
Fluoroquinolone-resistant	162	51 (31.5)	1.29 (1.00 to 1.67)	0.050
Extended-spectrum cephalosporin-resistant	293	83 (28.3)	1.15 (0.90 to 1.46)	0.264
Carbapenem-resistant	232	92 (39.7)	1.31 (1.04 to 1.64)	0.019
DTR	516	278 (53.9)	1.53 (1.27 to 1.84)	<0.001
Active first-line categories (N = 1443)				
Adequate: 3–5 active categories (reference)	580	137 (23.6)	1.00	–
Limited: 1–2 active categories	347	127 (36.6)	1.24 (1.02 to 1.51)	0.033
DTR: 0 active categories	516	278 (53.9)	1.46 (1.23 to 1.73)	<0.001

Modified Poisson models adjusted for age, sex, ICU at culture, potential BSI source, healthcare-associated infection, COVID-19 era, calendar year and Elixhauser Comorbidity Index. Active first-line categories counted carbapenems, extended-spectrum cephalosporins, fluoroquinolones, piperacillin-tazobactam when tested, and aztreonam for non-*Acinetobacter* species or ampicillin-sulbactam for *A. baumannii* when tested. DTR, difficult-to-treat resistance; GNB, gram-negative bacilli; RR, risk ratio.

In the five-level resistance hierarchy (N = 1663), DTR had the largest adjusted association with mortality (aRR 1.53; 95% CI 1.27 to 1.84). Carbapenem resistance was also associated with higher mortality (aRR 1.31; 95% CI 1.04 to 1.64), whereas FQR was borderline (aRR 1.29; 95% CI 1.00 to 1.67) and ESC-R was not statistically significant (aRR 1.15; 95% CI 0.90 to 1.46). Organism group did not significantly modify the resistance-mortality association.

A graded relationship was observed across active first-line antimicrobial categories (P for trend <0.001; N = 1443). Compared with isolates susceptible to three to five first-line categories, isolates with one to two active categories had higher mortality (aRR 1.24; 95% CI 1.02 to 1.51), and isolates with no active first-line categories had the highest mortality (aRR 1.46; 95% CI 1.23 to 1.73). Sensitivity analyses supported the direction of the primary DTR estimates; excluding ICU status from the model produced the largest shift, consistent with residual severity confounding (**Table 3**; **Figure 3**; **Supplementary Tables 10**-**12**, **Supplementary Figures 7**-**9**).

## 4 Discussion

In this 12-year culture-based surveillance cohort, the broad distribution of bloodstream GNB remained relatively stable, but the resistance profile changed in a clinically important direction. FQR and ESC-R among Enterobacterales declined, while carbapenem resistance and DTR increased, particularly among Enterobacterales. DTR was associated with higher 30-day in-hospital mortality in both Enterobacterales and non-fermenting GNB, and mortality increased as the number of active first-line antimicrobial categories decreased.

These findings are consistent with the global emphasis on carbapenem-resistant *K. pneumoniae*, carbapenem-resistant *A. baumannii*, *carbapenem-resistant P. aeruginosa and* Enterobacterales as major public-health threats (Antimicrobial Resistance Collaborators, 2022; World Health Organization, 2022, 2024). They also align with Saudi reports documenting carbapenem-resistant or carbapenemase-producing *K. pneumoniae* and non-fermenter resistance as active regional problems (Alghoribi et al., 2020; Alkhelaiwi et al., 2026; Hafiz et al., 2023; Shabi et al., 2025, 2026a, b; Somily et al., 2021). The local pattern, however, is not simply a uniform rise in all resistance. The decline in FQR and ESC-R alongside increasing carbapenem resistance suggests that resistance surveillance should avoid relying on a single broad marker. At least in this setting, carbapenem resistance and DTR provided a sharper signal of constrained treatment options than earlier hierarchy levels.

The 30-day in-hospital mortality observed in this cohort (36.5%) is higher than the crude mortality typically reported across unselected gram-negative bacteraemia, including in the large 173-hospital US cohort of Kadri et al. (2018), yet is comparable to the crude mortality that same cohort reported specifically among difficult-to-treat resistant isolates and to the mortality reported among predominantly gram-negative, ICU-acquired bloodstream infections in the international EUROBACT-2 cohort (Tabah et al., 2023). This high value most likely reflects the ICU-predominant, healthcare-associated and resistance-enriched case-mix of the present cohort—paralleling the substantial case-fatality reported in regional Saudi *Klebsiella pneumoniae* bacteraemia (Hafiz et al., 2023)—rather than a generalized excess mortality risk across all gram-negative bacteraemia.

In our cohort, 30-day mortality was modestly higher during the COVID-19 era in unadjusted analysis but was no longer independently associated with death after adjustment for organism-resistance category and the other covariates (adjusted risk ratio 1.12; 95% CI 0.91 to 1.38), suggesting that the pandemic-era difference was largely explained by shifts in case-mix and resistance rather than a direct period effect. This is consistent with international reports describing pandemic-associated increases in carbapenem-resistant *Acinetobacter baumannii* and Enterobacterales bloodstream infections and a greater bloodstream-infection burden among critically ill patients, attributed to altered case-mix, intensive-care crowding, and disruption of infection-prevention and antimicrobial-stewardship practices during COVID-19 (O’Toole, 2021; Abubakar et al., 2023; Abdelmoneim et al., 2024).

The contrast between declining FQR and ESC-R but rising carbapenem resistance also mirrors a wider surveillance challenge: resistance indicators can move in different directions when antimicrobial use, clonal expansion and testing practices change over time. Reports from Saudi Arabia and other hospital settings emphasize carbapenem-resistant non-fermenting gram-negative bacilli as persistent clinical problems (Shabi et al., 2025; Somily et al., 2021). Our findings extend that context by linking these phenotypes to admission-level outcomes and by showing that DTR and active first-line category counts provide clinically useful stratification beyond organism group alone.

The mortality analysis should be interpreted as an adjusted association, not as proof that resistance caused death. Patients with DTR infections may have differed in unmeasured severity, source control, prior antimicrobial exposure, device use and timing of active therapy. The staged models support this caution: excluding ICU status from sensitivity models produced the largest shift in DTR estimates. Even with that limitation, the consistency across the organism-resistance composite, the resistance hierarchy and the active-agent categories strengthens the inference that DTR identifies a high-risk clinical subgroup.

The study has several strengths. It used exact study dates across a long surveillance period, excluded duplicate same-species isolate-episodes, distinguished isolate-level trend analyses from admission-level mortality analyses, modelled temporal change with segmented regression rather than independent year-by-year testing, evaluated non-linearity in continuous covariates, used risk ratios rather than odds ratios for a common outcome, and examined sensitivity to alternative mortality definitions and culture-selection rules.

The limitations are also important. This was a single-center retrospective study from a tertiary referral hospital, so external validity is limited. In this cohort, just over half of patients (50.8%) were managed in an intensive care unit and more than two-thirds (68.2%) had healthcare-associated infection, so the case mix skewed toward more acute, device-exposed and healthcare-exposed patients; these findings may not generalize to community-onset infections, non-ICU settings, or hospitals and regions with different levels of care, patient populations, or resistance epidemiology. The cohort was culture-based and not independently adjudicated as clinical bloodstream infection or sepsis; source attribution used concordant cultures as a proxy. Severity scores, treatment timing, source-control data and post-discharge mortality were unavailable. Specifically, illness-severity measures such as the SOFA score, the APACHE II score and the Pitt bacteremia score (Chow et al., 1991), together with data on septic shock, vasopressor requirement, empirical therapy adequacy, time to effective or active antimicrobial therapy, definitive therapy selection and source-control interventions, were not available. Because DTR is plausibly linked to both greater underlying illness severity and to delayed or inadequate treatment, the absence of these variables likely contributed to residual confounding in the adjusted mortality estimates; ICU status and the Elixhauser comorbidity index capture only part of this severity domain and cannot substitute for direct severity scoring or treatment-timing data. This interpretation is consistent with the sensitivity analysis, in which removing ICU status produced the largest shift in the DTR estimate, a pattern more suggestive of unmeasured severity confounding than of a stable causal effect. In addition, outcome ascertainment was restricted to 30-day in-hospital mortality, so deaths occurring after discharge but within the 30-day window were not captured; because hospital discharge functions as a competing event for death (Noordzij et al., 2013), this design likely underestimates true 30-day mortality and may do so differentially across resistance groups. Resistance mechanisms were not genetically confirmed, and breakpoint changes over time could not be fully standardized because raw MICs were unavailable. Complete-case susceptibility analyses may also have introduced bias if testing completeness was related to organism, period or patient severity.

A related limitation is that the high carbapenem resistance and rising difficult-to-treat resistance observed in *Klebsiella pneumoniae* and other Enterobacterales were defined phenotypically; no molecular characterization of carbapenemase genes (e.g., KPC, OXA-48-like, NDM, VIM, IMP) was performed. This distinction matters clinically, because KPC, OXA-48-like and the metallo-β-lactamases (NDM, VIM, IMP) differ in epidemiology, infection-control implications and treatment options, since susceptibility to newer β-lactam/β-lactamase-inhibitor agents (e.g., ceftazidime-avibactam, meropenem-vaborbactam) depends on the specific carbapenemase class present. Regional evidence indicates that the Saudi and wider Arabian Gulf carbapenemase landscape has historically been dominated by OXA-48-like and NDM enzymes, with emerging KPC producers increasingly reported (Alghoribi et al., 2020; Alkhelaiwi et al., 2026; Alshahrani et al., 2022; Shabi et al., 2026b). Nonetheless, DTR was defined using validated susceptibility criteria and remains a clinically actionable classification independent of the underlying genotype. Future prospective studies incorporating molecular surveillance of carbapenem-non-susceptible isolates are recommended to clarify local mechanism distribution and better inform targeted therapy and infection-control policy.

A further limitation concerns breakpoint evolution across the 12-year study period. CLSI carbapenem breakpoints for Enterobacterales were substantially lowered in the period predating this cohort, and cephalosporin/ESBL interpretive criteria were revised more than once between 2013 and 2024 (Clinical and Laboratory Standards Institute, 2026). Because raw MIC values were not retained, isolates could not be reinterpreted under a single, current standard; VITEK 2 software and antimicrobial card versions were also updated over time, a related source of methodological drift. In principle, this could inflate apparent early-period carbapenem and cephalosporin susceptibility relative to later years, contributing to or exaggerating the observed upward resistance trend. However, because the breakpoints in use at the time of testing were applied consistently at each time point rather than retrospectively, this bias is likely real but modest rather than the principal driver of the reported trends.

For practice, these data support local reporting that separates carbapenem resistance and DTR from broader non-susceptibility categories and makes the denominator explicit. Clinically, flagging DTR status at the time of preliminary organism identification—before full susceptibility results are available—could support earlier risk-stratified decisions, including reflex rapid diagnostic testing, more judicious empirical broadening in patients with risk factors for DTR organisms, and prioritized contact precautions, rather than waiting for conventional susceptibility panels to trigger these responses. For stewardship and infection prevention, the dominance of *K. pneumoniae* and the rise in DTR Enterobacterales point toward the importance of targeted review of empirical therapy pathways, rapid susceptibility reporting, device-associated infection prevention, source-control workflows and unit-specific feedback, especially in ICU settings. This fits with regional calls for stronger cross-institutional stewardship collaboration in Arab countries (Al Salman et al., 2021). Future work should link microbiology records to antimicrobial exposure, timing of active therapy, severity scores, molecular resistance mechanisms and post-discharge outcomes.

## 5 Conclusions

In this multiyear tertiary-hospital cohort in Southern Saudi Arabia, the composition of bloodstream gram-negative bacilli remained broadly stable, yet carbapenem resistance and difficult-to-treat resistance rose sharply, most strikingly in *K. pneumoniae*. DTR carried the strongest independent association with in-hospital mortality, following a graded dose-response as active first-line agents were lost. These findings indicate that escalating treatment-limiting resistance, rather than a shifting organism mix, is associated with worse outcomes. Surveillance must move beyond drug-by-drug reporting toward clinically interpretable treatment-option phenotypes linked to patient outcomes, and stewardship and infection-control efforts should prioritize curbing carbapenem resistance in Enterobacterales.

## Data Availability

The raw data supporting the conclusions of this article will be made available by the authors, without undue reservation.

## Supplementary material

The following **Supplementary Materials** accompany this article: the eMethods, **Supplementary Tables 1**-**14** and **Supplementary Figures 1**-**9**. The eMethods detail the CLSI-informed organism-antibiotic exclusion rules, the difficult-to-treat-resistance (DTR) derivation, the segmented (joinpoint) binomial regression model and weighted Bayesian information criterion model selection, the patient-clustered standard errors, and the subgroup-trend eligibility thresholds.

- **Supplementary Tables 1**-**14** provide the full comorbidity profile, the bloodstream-infection-source characteristics, the full species inventory by year, antimicrobial non-susceptibility by organism group and species, the segmented-trend model selection, the species-level trends, the linearity testing, the full multivariable mortality models, the resistance-hierarchy and active-agent models, the sensitivity analyses, the subgroup-trend analyses, and the trend-feasibility assessment.
- **Supplementary Figures 1**-**9** present the antimicrobial-susceptibility heatmaps, the species- and resistance-trend plots, the restricted-cubic-spline dose-response curves, the mortality forest plots, the staged-regression decomposition, and the sensitivity-analysis comparisons.
