# Supplementary material for: Temporal trends and mortality associated with difficult-to-treat resistance in bloodstream gram-negative bacilli: a retrospective cohort study in Southern Saudi Arabia
Source: Front Cell Infect Microbiol. 2026 Aug 31;16:1919929. doi: 10.3389/fcimb.2026.1919929 (PMC13572238; doi:10.3389/fcimb.2026.1919929)
Supplement: Supplementary file 1 [file SupplementaryFile1.docx]

**Supplementary Material**

# eMethod

## Study Population

Patients younger than 12 years were excluded because pediatric services were transferred from Aseer Central Hospital (ACH) to a dedicated pediatric hospital early in the study period; retaining these records would have introduced a structural artifact into temporal trend analyses.

## Microbiological Methods

Organisms were classified as Enterobacterales or non-fermenting gram-negative bacilli using taxonomic assignment and glucose fermentation status. Susceptibility results were harmonized to susceptible vs non-susceptible, with intermediate results grouped with resistant results to reduce misclassification from temporal breakpoint revisions. Organism-antibiotic pairs were excluded from descriptive susceptibility reporting and downstream phenotype derivation when the Clinical and Laboratory Standards Institute (CLSI) Appendix B identified intrinsic resistance, when organism-specific interpretive criteria were unavailable, or when the pair was prespecified as nonreportable in the bloodstream-study workflow (Clinical and Laboratory Standards Institute, 2026).

Moxifloxacin was excluded from organism-group comparisons and fluoroquinolone phenotype derivation because CLSI M100 does not provide interpretive breakpoints across the non-fermenting gram-negative bacilli spectrum used in those analyses. Amoxicillin was excluded because it was virtually untested for bloodstream isolates, and CLSI M100, 36th edition, provides urinary-only breakpoints for *Escherichia coli* and *Proteus mirabilis*. Amikacin for *Pseudomonas aeruginosa* was suppressed because CLSI M100, 36th edition (Table 1B-1), restricts amikacin breakpoints to urinary isolates only (revised in the 33rd edition, 2023). For non-fermenting gram-negative bacilli, first-generation cephalosporins, second-generation cephalosporins, and cephamycins were treated as nonreportable. Additional species-specific exclusions followed the reconciled CLSI M100, 36th edition, Appendix B matrix, including Proteeae tetracycline and tigecycline exclusions, *Providencia stuartii* gentamicin and tobramycin exclusions, and organism-specific aminopenicillin and cephalosporin exclusions among Enterobacterales (Clinical and Laboratory Standards Institute, 2026).

## Resistance Phenotype Definitions

Temporal trend analyses for Fluoroquinolone resistance (FQR) were restricted to Enterobacterales because Extended-spectrum cephalosporin resistance (ESC-R; the preceding hierarchy tier required for contextual interpretation, see below) is defined only for Enterobacterales; the all-GNB FQR prevalence is reported in the feasibility assessment (eTable 14). ESC-R was defined for Enterobacterales only as non-susceptibility to cefepime, ceftazidime, cefotaxime, or ceftriaxone. Carbapenem-resistant Enterobacterales were defined as Enterobacterales non-susceptible to at least 1 carbapenem. For non-Proteeae Enterobacterales, carbapenems included imipenem, meropenem, and ertapenem. For Proteeae (*Proteus*, *Providencia*, and *Morganella*), carbapenems included meropenem and ertapenem only because elevated imipenem minimum inhibitory concentrations (MICs) may reflect non-carbapenemase mechanisms under CLSI footnote d (Clinical and Laboratory Standards Institute, 2026). For non-fermenting gram-negative bacilli, carbapenem resistance in *Acinetobacter baumannii* and *Pseudomonas aeruginosa* was defined as non-susceptibility to imipenem or meropenem, with ertapenem not applicable (Clinical and Laboratory Standards Institute, 2026).

The mutually exclusive resistance hierarchy comprised non-resistant, FQR, ESC-R, carbapenem-resistant, and difficult-to-treat resistance (DTR) categories. The 6-level organism-resistance composite combined organism group with carbapenem resistance and DTR status. DTR was defined using organism-specific criteria adapted from Kadri et al. (2018) and was evaluated only when at least 1 carbapenem, 1 extended-spectrum cephalosporin, and 1 fluoroquinolone had been tested. For Enterobacterales, DTR required non-susceptibility to all tested carbapenems, all tested extended-spectrum cephalosporins, and all tested fluoroquinolones, with piperacillin-tazobactam and aztreonam contributing when tested; for Proteeae, the carbapenem component used meropenem and ertapenem only. For *P. aeruginosa*, DTR required non-susceptibility to imipenem and meropenem, ceftazidime and cefepime, ciprofloxacin and levofloxacin, and to piperacillin-tazobactam and aztreonam when tested. For *A. baumannii*, DTR required non-susceptibility to imipenem and meropenem, extended-spectrum cephalosporins, ciprofloxacin and levofloxacin, and to ampicillin-sulbactam and piperacillin-tazobactam when tested.

*Stenotrophomonas maltophilia* was excluded from primary DTR, the resistance hierarchy, and first-line active-agent derivation because the original Kadri framework did not include this organism and the standard 5-category first-line structure was not applicable. *Burkholderia cepacia* complex (n = 11) was also excluded from standard phenotype derivation because the available CLSI breakpoint framework was insufficient for routine breakpoint-based classification in this study workflow. The first-line active-agent count used the same organism-specific category set as the DTR definition and was grouped as 3 to 5 active categories, 1 to 2 active categories, or 0 active categories.

## Statistical Methods

### Segmented Binomial Regression Model

The annual probability of each binary outcome (organism proportions, species proportions, and resistance phenotypes) for isolate $i$ in calendar year $t$ was modeled using a piecewise log-linear formulation with a binomial distribution and log link:

$$\log\left( p_{t} \right)= \beta_{0}+\beta_{1}t+\sum_{j=1}^{k} \delta_{j}\left( t-\tau_{j} \right)$$

where $p_{it}$ is the probability of the outcome, $\beta_{1}$ is the baseline slope (log risk ratio per year), $\kappa$ is the number of joinpoints (0, 1, or 2), $\tauⱼ$ are the joinpoint locations (calendar years), $\deltaⱼ$ are the change-in-slope parameters at each joinpoint, and $\left( t - \tauⱼ \right)^{+}=\max\left( t - \tauⱼ, 0 \right)$ is the positive-part function ensuring slope changes apply only beyond the joinpoint. The within-segment slope for segment $s$ is $\beta^{1}+ \Sigma_{\left\{ j\leq s \right\}}\deltaⱼ$, from which the annual percentage change (APC) is derived as:

$$APC=100\left( e^{\beta_{s}}-1 \right)\%$$

Over a user-specified interval $\left[ a, b \right]$, the average APC (AAPC) is the length-weighted average of segment slopes:

$$AAPC=100\left( \exp\left( \sum_{s} w_{s}\beta_{s} \right)-1 \right)\%, \mathrm{where}w_{s}=\frac{\mathrm{years} \mathrm{in} \left[ a, b \right] \cap segments s}{b-a}$$

Confidence intervals for APC and AAPC were derived using the delta method (Clegg et al., 2009). For the difference in AAPC (ΔAAPC) between 2 independent strata, analytic confidence intervals used the National Cancer Institute (NCI) Joinpoint variance formulation (National Cancer Institute, 2024).

### Jointpoint Selection

Candidate models with $\kappa$ = 0, 1, or 2 joinpoints were fit via grid search over allowable year positions, constrained to a minimum of 2 years from study boundaries (2013, 2024) and 2 years between breakpoints. The final $\kappa$ was selected using the weighted Bayesian information criterion (WBIC) described by Kim et al. (2023), with comparability considerations informed by prior segmented-regression work from the same group (Kim et al., 2004). Patient-clustered robust standard errors were used throughout to account for multiple isolates per person.

### Covariable Preprocessing and Staged Mortality Modeling

Continuous covariables (age and Elixhauser Comorbidity Index) were winsorized at the 1st and 99th percentiles and z-standardized (mean = 0, standard deviation [SD] = 1). Linearity was assessed by comparing linear, restricted cubic spline (RCS), and fractional polynomial (FP) specifications using Akaike information criterion (AIC) differences greater than 2 and Wald tests (*P* < .05). Age and calendar year were modeled as RCS with 3 knots, and the Elixhauser index was retained as linear. To decompose confounding pathways, staged regression added covariable blocks sequentially: stage 1 included demographics (age and sex), comorbidity (Elixhauser Comorbidity Index), healthcare-associated infection, COVID-19 era, and calendar year; stage 2 added intensive care unit (ICU) status at culture; and stage 3 added identified bloodstream infection source. The primary organism-resistance composite model had an AIC of 2,079.0.

### Subgroup Trend Eligibility

Subgroup trend analyses by sex (men vs women) and by ICU setting (ICU vs non-ICU) were conducted for species proportions and resistance phenotypes. Each stratification was gated independently, so an outcome could qualify for the sex comparison but not the ICU comparison, or vice versa. Trend analyses were restricted to outcomes meeting prespecified feasibility criteria: at least 50 events, at least 100 evaluable isolates, and an outcome prevalence between 0.5% and 99.5%. These thresholds were informed by simulation evidence showing that logistic-family models require approximately 10 to 20 events per parameter to avoid bias (Peduzzi et al., 1996; Vittinghoff and McCulloch, 2007). Because each segmented regression model contained 2 to 4 parameters, 50 events provided an events-per-parameter ratio of at least 12.5, exceeding the minimum recommended by Vittinghoff and McCulloch (2007). The non-ICU stratum (n = 928 isolates, 45.8% of the isolate-level cohort) was the binding constraint for most outcomes. The complete feasibility assessment, including event counts per stratum and inclusion or exclusion decisions for each outcome, is reported in eTable 14.

Species-specific resistance phenotypes (carbapenem-resistant *K. pneumoniae*, *E. coli*, *A. baumannii*, *P. aeruginosa*; and DTR Enterobacterales, *K. pneumoniae*, *E. coli*, *A. baumannii*, and *P. aeruginosa*) were assessed independently for each comparison; most were excluded from 1 or both subgroup comparisons because the organism-specific denominator, when further split by sex or ICU setting, fell below the 50-event threshold in at least 1 stratum. For each included outcome, stratum-specific breakpoints were selected independently using the same WBIC procedure, allowing different joinpoint structures across strata. ΔAAPC was computed by subtracting women from men and non-ICU from ICU, with analytic confidence intervals obtained from the same NCI Joinpoint variance formulation used for the main trend models (National Cancer Institute, 2024).

# eReferences

Clegg, L. X., Hankey, B. F., Tiwari, R., Feuer, E. J., and Edwards, B. K. (2009). Estimating average annual per cent change in trend analysis. Stat. Med. 28, 3670–3682. doi: 10.1002/sim.3733

Clinical and Laboratory Standards Institute (2026). Performance Standards for Antimicrobial Susceptibility Testing, 36th Edn. CLSI supplement M100. Wayne, PA: CLSI.

Kadri, S. S., Adjemian, J., Lai, Y. L., Spaulding, A. B., Ricotta, E., Prevots, D. R., et al. (2018). Difficult-to-treat resistance in gram-negative bacteremia at 173 US hospitals: retrospective cohort analysis of prevalence, predictors, and outcome of resistance to all first-line agents. Clin. Infect. Dis. 67, 1803–1814. doi: 10.1093/cid/ciy378

Kim, H. J., Fay, M. P., Yu, B., Barrett, M. J., and Feuer, E. J. (2004). Comparability of segmented line regression models. Biometrics 60, 1005–1014. doi: 10.1111/j.0006-341X.2004.00256.x

Kim, H. J., Chen, H. S., Midthune, D., Wheeler, B., Buckman, D. W., Green, D., et al. (2023). Data-driven choice of a model selection method in joinpoint regression. J. Appl. Stat. 50, 1992–2013. doi: 10.1080/02664763.2022.2063265

National Cancer Institute (2024). Joinpoint Regression Program, Version 5.2.0. Bethesda, MD: Statistical Methodology and Applications Branch, Surveillance Research Program, National Cancer Institute.

Peduzzi, P., Concato, J., Kemper, E., Holford, T. R., and Feinstein, A. R. (1996). A simulation study of the number of events per variable in logistic regression analysis. J. Clin. Epidemiol. 49, 1373–1379. doi: 10.1016/s0895-4356(96)00236-3

Vittinghoff, E., and McCulloch, C. E. (2007). Relaxing the rule of ten events per variable in logistic and Cox regression. Am. J. Epidemiol. 165, 710–718. doi: 10.1093/aje/kwk052
