# Supplementary material for: Temporal trends and mortality associated with difficult-to-treat resistance in bloodstream gram-negative bacilli: a retrospective cohort study in Southern Saudi Arabia
Source: Front Cell Infect Microbiol. 2026 Aug 31;16:1919929. doi: 10.3389/fcimb.2026.1919929 (PMC13572238; doi:10.3389/fcimb.2026.1919929)
Supplement: Supplementary file 2 [file SupplementaryFile2.docx]

**Supplementary Materials**

**Supplementary Table 1.** Full Comorbidity Profile by Organism Group Among Bloodstream Gram-Negative Bacilli (N = 1,728) ᵃ

| Comorbidity | Total (N = 1,728) | Organism Group | |
| --- | --- | --- | --- |
|  |  | Enterobacterales (n = 1,254) | Non-fermenting GNB (n = 474) |
| Acute kidney injury ᵇ | 41 (2.4) | 30 (2.4) | 11 (2.3) |
| Asthma ᵇ | 10 (0.6) | 6 (0.5) | 4 (0.8) |
| Atrial fibrillation ᵇ | 18 (1.0) | 14 (1.1) | 4 (0.8) |
| Autoimmune/connective tissue diseases | 66 (3.8) | 52 (4.1) | 14 (3.0) |
| Cardiac arrhythmias | 21 (1.2) | 17 (1.4) | 4 (0.8) |
| Chronic kidney disease ᵇ | 176 (10.2) | 131 (10.4) | 45 (9.5) |
| Chronic liver disease | 12 (0.7) | 10 (0.8) | 2 (0.4) |
| Chronic obstructive pulmonary disease ᵇ | 20 (1.2) | 12 (1.0) | 8 (1.7) |
| Chronic pulmonary disease | 39 (2.3) | 26 (2.1) | 13 (2.7) |
| Coagulopathy | 1 (0.1) | 1 (0.1) | 0 (0.0) |
| Congestive heart failure | 37 (2.1) | 26 (2.1) | 11 (2.3) |
| Deep vein thrombosis ᵇ | 29 (1.7) | 24 (1.9) | 5 (1.1) |
| Deficiency anemia | 7 (0.4) | 1 (0.1) | 6 (1.3) |
| Dementia ᵇ | 22 (1.3) | 18 (1.4) | 4 (0.8) |
| Depression | 5 (0.3) | 4 (0.3) | 1 (0.2) |
| Diabetes mellitus | 304 (17.6) | 242 (19.3) | 62 (13.1) |
| Complicated | 76 (4.4) | 60 (4.8) | 16 (3.4) |
| Uncomplicated | 228 (13.2) | 182 (14.5) | 46 (9.7) |
| End-stage kidney disease ᵇ | 149 (8.6) | 108 (8.6) | 41 (8.6) |
| Epilepsy ᵇ | 22 (1.3) | 14 (1.1) | 8 (1.7) |
| Fluid/electrolyte disorders | 4 (0.2) | 3 (0.2) | 1 (0.2) |
| HIV/AIDS | 1 (0.1) | 1 (0.1) | 0 (0.0) |
| Hypertension | 693 (40.1) | 522 (41.6) | 171 (36.1) |
| Complicated | 234 (13.5) | 172 (13.7) | 62 (13.1) |
| Uncomplicated | 459 (26.6) | 350 (27.9) | 109 (23.0) |
| Hypothyroidism | 57 (3.3) | 40 (3.2) | 17 (3.6) |
| Ischemic heart disease ᵇ | 57 (3.3) | 43 (3.4) | 14 (3.0) |
| Malignancy (any) | 189 (10.9) | 152 (12.1) | 37 (7.8) |
| Lymphoma/leukemia | 19 (1.1) | 16 (1.3) | 3 (0.6) |
| Metastatic cancer | 22 (1.3) | 19 (1.5) | 3 (0.6) |
| Solid tumor without metastasis | 148 (8.6) | 117 (9.3) | 31 (6.5) |
| Myocardial infarction ᵇ | 22 (1.3) | 15 (1.2) | 7 (1.5) |
| Obesity | 7 (0.4) | 4 (0.3) | 3 (0.6) |
| Other neurological disorders | 34 (2.0) | 22 (1.8) | 12 (2.5) |
| Paralysis | 3 (0.2) | 3 (0.2) | 0 (0.0) |
| Peptic ulcer disease | 0 (0.0) | 0 (0.0) | 0 (0.0) |
| Peripheral vascular disease | 28 (1.6) | 19 (1.5) | 9 (1.9) |
| Psychoses | 4 (0.2) | 4 (0.3) | 0 (0.0) |
| Pulmonary circulation disorders | 24 (1.4) | 15 (1.2) | 9 (1.9) |
| Pulmonary embolism ᵇ | 24 (1.4) | 15 (1.2) | 9 (1.9) |
| Renal failure | 212 (12.3) | 157 (12.5) | 55 (11.6) |
| Rheumatoid arthritis ᵇ | 0 (0.0) | 0 (0.0) | 0 (0.0) |
| Sickle cell disease ᵇ | 15 (0.9) | 8 (0.6) | 7 (1.5) |
| Stroke ᵇ | 151 (8.7) | 113 (9.0) | 38 (8.0) |
| Systemic lupus erythematosus ᵇ | 12 (0.7) | 11 (0.9) | 1 (0.2) |
| Valvular heart disease | 1 (0.1) | 1 (0.1) | 0 (0.0) |
| Weight loss | 1 (0.1) | 1 (0.1) | 0 (0.0) |
| Abbreviations: AIDS, acquired immunodeficiency syndrome; GNB, gram-negative bacilli; HIV, human immunodeficiency virus; N, total admissions; n, number with comorbidity. | | | |
| a—Restricted to the first bloodstream gram-negative bacilli culture per hospital admission (same cohort as Table 1).  b—Not included in the standard Elixhauser Comorbidity Index. | | | |

**Supplementary Table 2.** Characteristics of Bloodstream Gram-Negative Bacilli Episodes by BSI Source Status (N = 1,728) ᵃ

| **Characteristic** | **Total**  **(N = 1,728)** | **BSI Source Status ᵇ** | |
| --- | --- | --- | --- |
|  |  | **No Source Identified**  **(n = 1,010)** | **Source Identified**  **(n = 718)** |
| Demographics | | | |
| Age, yrs, median (IQR) | 60 (39–74) | 60 (38–74) | 61 (40–75) |
| Women, n (%) | 595 (34.4) | 356 (35.2) | 239 (33.3) |
| Organism Group | | | |
| Non-fermenting GNB, n (%) | 474 (27.4) | 316 (31.3) | 158 (22.0) |
| Top Species | | | |
| Klebsiella pneumoniae, n (%) | 646 (37.4) | 269 (26.6) | 377 (52.5) |
| Escherichia coli, n (%) | 293 (17.0) | 174 (17.2) | 119 (16.6) |
| Acinetobacter baumannii, n (%) | 252 (14.6) | 137 (13.6) | 115 (16.0) |
| Pseudomonas aeruginosa, n (%) | 145 (8.4) | 104 (10.3) | 41 (5.7) |
| Proteus mirabilis, n (%) | 50 (2.9) | 27 (2.7) | 23 (3.2) |
| Clinical Setting | | | |
| Intensive care unit at culture, n (%) | 878 (50.8) | 438 (43.4) | 440 (61.3) |
| Healthcare-associated (>2 days), n (%) | 1,179 (68.2) | 623 (61.7) | 556 (77.4) |
| Length of stay, days, median (IQR) | 25 (12–48) | 21 (10–44) | 31 (16–53) |
| Days from admission to culture, median (IQR) | 10 (1–25) | 7 (0–22) | 13 (4–28) |
| Resistance Phenotype ᶜ | | | |
| Extended-spectrum cephalosporin-resistant, n/N (%) | 741/1,254 (59.1) | 361/694 (52.0) | 380/560 (67.9) |
| Carbapenem-resistant Enterobacterales, n/N (%) | 493/1,254 (39.3) | 198/694 (28.5) | 295/560 (52.7) |
| Carbapenem-resistance (Overall), n/N (%) | 748/1,611 (46.4) | 330/913 (36.1) | 418/698 (59.9) |
| Difficult-to-treat (Overall), n/N (%) | 516/1,443 (35.8) | 212/820 (25.9) | 304/623 (48.8) |
| COVID-19 era | | | |
| COVID-19 era (2020–2024), n (%) | 940 (54.4) | 528 (52.3) | 412 (57.4) |
| Comorbidity Burden | | | |
| Elixhauser Comorbidity Index, median (IQR) | 0 (0–4) | 0 (0–4) | 0 (0–2) |
| Outcome | | | |
| 30-day mortality, n (%) | 630 (36.5) | 336 (33.3) | 294 (40.9) |
| BSI Source Type (among source-identified cases) | | | |
| Respiratory | — | — | 381 (53.1) |
| Urinary | — | — | 228 (31.8) |
| Wound | — | — | 69 (9.6) |
| Other | — | — | 40 (5.6) |
| Abbreviations: BSI, bloodstream infection; COVID, coronavirus disease 2019; GNB, gram-negative bacilli; IQR, interquartile range; n, number with characteristic; N, number evaluable. | | | |
| a—Admission-level cohort: one culture per admission (first bloodstream gram-negative bacilli [GNB] episode), yielding 1,728 admission records from 1,695 unique patients (drawn from 2,027 total isolate-episodes).  b—Bloodstream infection (BSI) source identified: concordant non-blood culture of same patient, same species within ±7 days of blood culture.  c—Resistance denominators vary by phenotype: Carbapenem-resistant Enterobacterales and Extended-spectrum cephalosporin-resistant are restricted to Enterobacterales only; other phenotypes include all GNB. | | | |

**Supplementary Table 3.** Full Species Inventory by Year Among Bloodstream Gram-Negative Bacilli, 2013–2024 (N = 2,027)

| Species | 2013 | 2014 | 2015 | 2016 | 2017 | 2018 | 2019 | 2020 | 2021 | 2022 | 2023 | 2024 | Total | % |
| --- | --- | --- | --- | --- | --- | --- | --- | --- | --- | --- | --- | --- | --- | --- |
| Enterobacterales | | | | | | | | | | | | | | |
| *Citrobacter braakii* | — | 1 | — | — | — | — | — | — | 1 | — | 1 | — | 3 | 0.1 |
| *Citrobacter freundii* | 3 | — | 1 | — | — | — | — | — | — | — | 1 | — | 5 | 0.2 |
| *Citrobacter koseri* | — | — | 1 | — | 1 | — | — | — | — | — | — | — | 2 | 0.1 |
| *Citrobacter sp.* | — | — | — | — | — | — | 1 | 1 | 3 | — | — | 1 | 6 | 0.3 |
| *Enterobacter cloacae* | 11 | 5 | 2 | 8 | 4 | 6 | 10 | 3 | 9 | 15 | 8 | 2 | 83 | 4.1 |
| *Enterobacter hormaechei* | — | — | — | — | — | — | — | — | — | 1 | — | — | 1 | 0.0 |
| *Enterobacter sp.* | 1 | — | — | — | — | — | — | — | — | — | — | — | 1 | 0.0 |
| *Escherichia coli* | 30 | 19 | 13 | 19 | 13 | 26 | 19 | 26 | 28 | 53 | 35 | 34 | 315 | 15.5 |
| *Klebsiella aerogenes* | 3 | 3 | 4 | 3 | 1 | 3 | 1 | — | 4 | 1 | 5 | 6 | 34 | 1.7 |
| *Klebsiella ornithinolytica* | — | — | — | — | — | — | — | 1 | — | — | — | — | 1 | 0.0 |
| *Klebsiella oxytoca* | — | 1 | — | — | — | — | 2 | 2 | — | 2 | 4 | 2 | 13 | 0.6 |
| *Klebsiella pneumoniae* | 33 | 21 | 14 | 51 | 31 | 38 | 90 | 107 | 155 | 117 | 62 | 43 | 762 | 37.6 |
| *Klebsiella pneumoniae subsp. ozaenae* | — | — | — | — | — | — | 1 | — | — | — | — | — | 1 | 0.0 |
| *Klebsiella sp.* | 1 | — | — | — | — | — | — | — | — | — | — | — | 1 | 0.0 |
| *Lelliottia amnigena* | — | 1 | — | — | — | — | — | — | — | — | — | — | 1 | 0.0 |
| *Morganella morganii* | 1 | 3 | 4 | 1 | 1 | 2 | 1 | — | 1 | — | 1 | — | 15 | 0.7 |
| *Pantoea agglomerans* | — | 1 | 1 | 1 | — | — | — | — | — | — | — | — | 3 | 0.1 |
| *Proteus mirabilis* | 12 | 3 | 7 | 15 | 8 | 6 | 5 | 3 | 3 | 6 | 2 | 1 | 71 | 3.5 |
| *Proteus penneri* | 1 | — | — | — | — | — | — | — | — | — | — | — | 1 | 0.0 |
| *Proteus sp.* | 3 | — | — | — | — | — | — | — | — | — | — | — | 3 | 0.1 |
| *Proteus vulgaris* | — | — | 1 | — | — | — | — | — | — | — | — | — | 1 | 0.0 |
| *Providencia rettgeri* | 1 | — | — | — | — | — | — | — | — | — | — | — | 1 | 0.0 |
| *Providencia rustigianii* | — | 1 | — | — | — | — | — | — | — | — | — | — | 1 | 0.0 |
| *Providencia sp.* | 1 | — | — | — | — | — | — | — | — | — | — | — | 1 | 0.0 |
| *Providencia stuartii* | 5 | 6 | 9 | 4 | 2 | 1 | 2 | 1 | 3 | 6 | 1 | — | 40 | 2.0 |
| *Salmonella enterica paratyphi A* | — | — | — | — | 1 | — | — | — | — | — | — | — | 1 | 0.0 |
| *Salmonella sp.* | — | 3 | — | 2 | 2 | 1 | 2 | 2 | — | — | 1 | 3 | 16 | 0.8 |
| *Salmonella typhi* | — | — | — | — | — | — | — | 1 | — | — | 1 | — | 2 | 0.1 |
| *Serratia ficaria* | — | 1 | — | — | — | — | — | — | — | — | — | — | 1 | 0.0 |
| *Serratia liquefaciens* | 1 | 2 | — | — | 1 | — | — | — | — | — | — | — | 4 | 0.2 |
| *Serratia marcescens* | 1 | — | 5 | 17 | — | 3 | 10 | 7 | 6 | 8 | 8 | 29 | 94 | 4.6 |
| *Serratia plymuthica* | — | — | 1 | — | — | — | — | — | — | — | — | — | 1 | 0.0 |
| *Serratia rubidaea* | — | — | — | — | 1 | 1 | — | — | — | — | — | — | 2 | 0.1 |
| Subtotal | 108 | 71 | 63 | 121 | 66 | 87 | 144 | 154 | 213 | 209 | 130 | 121 | 1,487 | 73.4 |
| Non-fermenting GNB | | | | | | | | | | | | | | |
| *Achromobacter sp.* | — | — | 1 | 4 | — | — | — | — | — | — | — | 1 | 6 | 0.3 |
| *Acinetobacter baumannii* | 13 | 14 | 25 | 32 | 21 | 18 | 28 | 42 | 22 | 27 | 22 | 13 | 277 | 13.7 |
| *Acinetobacter lwoffii* | — | — | — | 1 | — | — | — | — | 1 | 4 | — | — | 6 | 0.3 |
| *Acinetobacter sp.* | 1 | — | — | — | — | — | — | — | — | — | — | 1 | 2 | 0.1 |
| *Alcaligenes sp.* | — | 1 | — | — | — | — | — | — | — | — | — | — | 1 | 0.0 |
| *Bordetella bronchiseptica* | — | — | — | — | — | — | 1 | — | — | — | — | — | 1 | 0.0 |
| *Burkholderia cepacia* | — | — | — | 1 | — | — | — | 3 | 5 | — | — | 2 | 11 | 0.5 |
| *Burkholderia gladioli* | — | — | — | — | 1 | — | — | — | — | 1 | — | — | 2 | 0.1 |
| *Moraxella sp.* | 1 | — | — | — | — | — | — | — | — | — | — | — | 1 | 0.0 |
| *Pseudomonas aeruginosa* | 18 | 6 | 14 | 19 | 15 | 19 | 14 | 18 | 11 | 18 | 14 | 10 | 176 | 8.7 |
| *Pseudomonas alcaligenes* | — | — | — | — | — | — | — | — | 1 | — | — | — | 1 | 0.0 |
| *Pseudomonas fluorescens* | — | 1 | — | — | — | — | 2 | — | — | — | 1 | — | 4 | 0.2 |
| *Pseudomonas luteola* | — | — | — | 1 | — | — | — | — | — | — | — | — | 1 | 0.0 |
| *Pseudomonas sp.* | — | — | — | — | 1 | — | 1 | 3 | 1 | — | — | 1 | 7 | 0.3 |
| *Ralstonia pickettii* | — | — | 1 | — | — | — | — | — | — | — | — | — | 1 | 0.0 |
| *Stenotrophomonas maltophilia* | — | 1 | 3 | 11 | 3 | — | 1 | 7 | 6 | 2 | 1 | 6 | 41 | 2.0 |
| *Stutzerimonas stutzeri* | — | — | 1 | 1 | — | — | — | — | — | — | — | — | 2 | 0.1 |
| Subtotal | 33 | 23 | 45 | 70 | 41 | 37 | 47 | 73 | 47 | 52 | 38 | 34 | 540 | 26.6 |
| Total | 141 | 94 | 108 | 191 | 107 | 124 | 191 | 227 | 260 | 261 | 168 | 155 | 2,027 | 100.0 |
| Abbreviations: GNB, gram-negative bacilli. | | | | | | | | | | | | | | |

**Supplementary Table 4.** Antimicrobial Non-Susceptibility Rates by Organism Group Among Bloodstream Gram-Negative Bacilli (N = 2,027) ᵃ

| Antibiotic | Total n/N (%) | Organism Group | | P value |
| --- | --- | --- | --- | --- |
|  |  | Enterobacterales n/N (%) | Non-fermenting GNB n/N (%) |  |
| Aminoglycosides | | | | |
| Amikacin | 696/1633 (42.6) | 555/1420 (39.1) | 141/213 (66.2) | <0.001 |
| Gentamicin | 822/1677 (49.0) | 629/1379 (45.6) | 193/298 (64.8) | <0.001 |
| Tobramycin | 605/1188 (50.9) | 481/878 (54.8) | 124/310 (40.0) | <0.001 |
| Carbapenems | | | | |
| Ertapenem | 407/929 (43.8) | 407/929 (43.8) | — | — |
| Imipenem | 772/1788 (43.2) | 485/1361 (35.6) | 287/427 (67.2) | <0.001 |
| Meropenem | 776/1843 (42.1) | 504/1398 (36.1) | 272/445 (61.1) | <0.001 |
| Cephalosporins | | | | |
| Cefazolin | 50/144 (34.7) | 50/144 (34.7) | — | — |
| Cefepime | 1057/1685 (62.7) | 765/1229 (62.2) | 292/456 (64.0) | 0.533 |
| Cefotaxime | 364/548 (66.4) | 315/485 (64.9) | 49/63 (77.8) | 0.047 |
| Cefoxitin | 341/649 (52.5) | 341/649 (52.5) | — | — |
| Ceftazidime | 1058/1635 (64.7) | 768/1181 (65.0) | 290/454 (63.9) | 0.686 |
| Ceftriaxone | 249/330 (75.5) | 211/290 (72.8) | 38/40 (95.0) | 0.001 |
| Cefuroxime | 400/530 (75.5) | 400/530 (75.5) | — | — |
| Fluoroquinolones | | | | |
| Ciprofloxacin | 1186/1880 (63.1) | 914/1421 (64.3) | 272/459 (59.3) | 0.052 |
| Levofloxacin | 869/1486 (58.5) | 669/1090 (61.4) | 200/396 (50.5) | <0.001 |
| Penicillin + β-Lactamase Inhibitor | | | | |
| Amoxicillin-clavulanate | 610/937 (65.1) | 610/937 (65.1) | — | — |
| Ampicillin-sulbactam | 606/790 (76.7) | 495/642 (77.1) | 111/148 (75.0) | 0.591 |
| Piperacillin-tazobactam | 814/1626 (50.1) | 653/1350 (48.4) | 161/276 (58.3) | 0.003 |
| Tetracyclines | | | | |
| Minocycline | 94/183 (51.4) | 84/151 (55.6) | 10/32 (31.2) | 0.019 |
| Tigecycline | 185/1078 (17.2) | 185/1077 (17.2) | — | — |
| Other | | | | |
| Ampicillin | 167/210 (79.5) | 167/210 (79.5) | — | — |
| Aztreonam | 456/736 (62.0) | 419/657 (63.8) | 37/79 (46.8) | 0.005 |
| Trimethoprim-sulfamethoxazole | 800/1470 (54.4) | 788/1410 (55.9) | 12/60 (20.0) | <0.001 |
| Abbreviations: GNB, gram-negative bacilli; n, number non-susceptible; N, number tested. | | | | |
| a—Denominators vary by antibiotic: species-antibiotic combinations were excluded when Clinical and Laboratory Standards Institute (CLSI) interpretive criteria were not reportable for that organism or when combinations were suppressed by CLSI-informed project rules (CLSI M100-Ed36 Appendix B). | | | | |

**Supplementary Table 5.** Species-Specific Antimicrobial Non-Susceptibility Rates Among Bloodstream Gram-Negative Bacilli ᵃ

| Antibiotic | Enterobacterales | | | | | |  | Non-fermenting GNB | |
| --- | --- | --- | --- | --- | --- | --- | --- | --- | --- |
|  | *E. cloacae* | *E. coli* | *K. pneumoniae* | *M. morganii* | *P. mirabilis* | *P. stuartii* |  | *A. baumannii* | *P. aeruginosa* |
| Aminoglycosides | | | | | | | | | |
| Amikacin | 6/82 (7.3) | 32/302 (10.6) | 431/737 (58.5) | 2/15 (13.3) | 41/68 (60.3) | 7/39 (17.9) |  | 132/183 (72.1) | NR |
| Gentamicin | 18/82 (22.0) | 77/302 (25.5) | 424/725 (58.5) | 7/15 (46.7) | 59/70 (84.3) | NR |  | 184/269 (68.4) | NR |
| Tobramycin | 9/53 (17.0) | 75/195 (38.5) | 339/465 (72.9) | 1/6 (16.7) | 26/30 (86.7) | NR |  | 93/179 (52.0) | 26/112 (23.2) |
| Carbapenems | | | | | | | | | |
| Ertapenem | 11/55 (20.0) | 12/197 (6.1) | 359/500 (71.8) | 0/6 (0.0) | 4/38 (10.5) | 2/17 (11.8) |  | NR | NR |
| Imipenem | 9/79 (11.4) | 10/299 (3.3) | 398/719 (55.4) | 5/13 (38.5) | 13/52 (25.0) | 14/38 (36.8) |  | 216/236 (91.5) | 62/164 (37.8) |
| Meropenem | 5/80 (6.2) | 9/295 (3.1) | 450/722 (62.3) | 1/14 (7.1) | 6/64 (9.4) | 4/38 (10.5) |  | 211/257 (82.1) | 52/158 (32.9) |
| Cephalosporins | | | | | | | | | |
| Cefepime | 25/73 (34.2) | 106/214 (49.5) | 507/661 (76.7) | 5/15 (33.3) | 41/53 (77.4) | 30/39 (76.9) |  | 223/263 (84.8) | 56/162 (34.6) |
| Ceftriaxone | 6/12 (50.0) | 38/56 (67.9) | 142/174 (81.6) | 1/3 (33.3) | 10/14 (71.4) | 6/6 (100.0) |  | 36/38 (94.7) | NR |
| Cefotaxime | 23/41 (56.1) | 26/72 (36.1) | 227/291 (78.0) | 3/5 (60.0) | 7/11 (63.6) | 19/22 (86.4) |  | 45/54 (83.3) | NR |
| Ceftazidime | 36/74 (48.6) | 107/203 (52.7) | 484/627 (77.2) | 7/14 (50.0) | 40/52 (76.9) | 30/38 (78.9) |  | 221/260 (85.0) | 56/166 (33.7) |
| Cefuroxime | 28/37 (75.7) | 40/79 (50.6) | 287/353 (81.3) | NR | 10/14 (71.4) | 15/18 (83.3) |  | NR | NR |
| Cefazolin | NT | 15/48 (31.2) | 26/82 (31.7) | NR | 5/7 (71.4) | NR |  | NR | NR |
| Cefoxitin | NR | 22/168 (13.1) | 299/410 (72.9) | 3/3 (100.0) | 3/30 (10.0) | 4/19 (21.1) |  | NR | NR |
| Fluoroquinolones | | | | | | | | | |
| Ciprofloxacin | 21/81 (25.9) | 190/302 (62.9) | 545/728 (74.9) | 11/14 (78.6) | 60/69 (87.0) | 31/35 (88.6) |  | 219/262 (83.6) | 43/167 (25.7) |
| Levofloxacin | 12/64 (18.8) | 145/227 (63.9) | 404/554 (72.9) | 8/12 (66.7) | 45/50 (90.0) | 20/27 (74.1) |  | 154/208 (74.0) | 34/122 (27.9) |
| Penicillin + β-Lactamase Inhibitor | | | | | | | | | |
| Amoxicillin-clavulanate | NR | 104/245 (42.4) | 458/589 (77.8) | NR | 21/56 (37.5) | NR |  | NR | NR |
| Ampicillin-sulbactam | NR | 111/167 (66.5) | 342/420 (81.4) | 3/3 (100.0) | 20/23 (87.0) | 8/11 (72.7) |  | 109/143 (76.2) | NR |
| Piperacillin-tazobactam | 24/76 (31.6) | 47/292 (16.1) | 511/713 (71.7) | 1/14 (7.1) | 9/64 (14.1) | 9/39 (23.1) |  | 83/97 (85.6) | 68/156 (43.6) |
| Tetracyclines | | | | | | | | | |
| Minocycline | 2/9 (22.2) | 14/34 (41.2) | 65/95 (68.4) | 1/1 (100.0) | NR | NR |  | 10/31 (32.3) | NR |
| Tigecycline | 7/59 (11.9) | 4/239 (1.7) | 153/628 (24.4) | NR | NR | NR |  | NR | NR |
| Other | | | | | | | | | |
| Ampicillin | NR | 123/152 (80.9) | NR | NR | 26/32 (81.2) | NR |  | NR | NR |
| Aztreonam | 26/41 (63.4) | 45/95 (47.4) | 293/382 (76.7) | 1/4 (25.0) | 12/20 (60.0) | 8/11 (72.7) |  | NR | 32/71 (45.1) |
| Trimethoprim-sulfamethoxazole | 25/78 (32.1) | 155/299 (51.8) | 459/721 (63.7) | 9/14 (64.3) | 58/67 (86.6) | 34/39 (87.2) |  | NT | NR |
| Abbreviations: GNB, gram-negative bacilli; NR, not reported (no validated Clinical and Laboratory Standards Institute [CLSI] interpretive criteria for that species-antibiotic combination or suppressed by CLSI-informed project rules [CLSI M100-Ed36 Appendix B]); NT, not tested (antibiotic not performed by the laboratory for this species). Species: A. baumannii, Acinetobacter baumannii; E. cloacae, Enterobacter cloacae; E. coli, Escherichia coli; K. pneumoniae, Klebsiella pneumoniae; M. morganii, Morganella morganii; P. aeruginosa, Pseudomonas aeruginosa; P. mirabilis, Proteus mirabilis; P. stuartii, Providencia stuartii. | | | | | | | | | |
| a—Format: number non-susceptible / number tested (%). | | | | | | | | | |

 Supplementary Figure 1. Antimicrobial Non-Susceptibility Rates by Species and Drug Class Among Bloodstream Gram-Negative Bacilli. Cell values represent percentage of non-susceptible isolates. NR, no validated Clinical and Laboratory Standards Institute (CLSI) breakpoint or intrinsic resistance; NT, not tested. Abbreviations: Amox-clav, amoxicillin-clavulanate; Amp-sulb, ampicillin-sulbactam; BLI, beta-lactamase inhibitor; NFGNB, non-fermenting gram-negative bacilli; Pip-tazo, piperacillin-tazobactam; TMP-SMX, trimethoprim-sulfamethoxazole. A. baumannii, Acinetobacter baumannii; E. cloacae, Enterobacter cloacae; E. coli, Escherichia coli; K. pneumoniae, Klebsiella pneumoniae; M. morganii, Morganella morganii; P. aeruginosa, Pseudomonas aeruginosa; P. mirabilis, Proteus mirabilis; P. stuartii, Providencia stuartii.

**Supplementary Table 6.** Segmented Trend Model Selection and Average Annual Percentage Change (AAPC) Summary, 2013–2024

| Outcome | Breakpoint(s) ᵃ | Segments | WBIC ᵃ | AAPC (%/yr) ᵃ | 95% CI | Trend ᵇ |
| --- | --- | --- | --- | --- | --- | --- |
| Enterobacterales proportion | | | | | | |
| Overall | 2015 | 2 | 2361.8 | 0.5 | -0.3 to 1.3 | Stable |
| ICU | Linear | 1 | 1307.2 | 1.1 | -0.1 to 2.4 | Stable |
| Non-ICU | Linear | 1 | 1078.4 | 2.4 | 1.2 to 3.6 | Increasing |
| Non-fermenting GNB proportion | | | | | | |
| Overall | 2015 | 2 | 2358.6 | -1.4 | -4.3 to 1.4 | Stable |
| ICU | Linear | 1 | 1307.4 | -2.7 | -5.6 to 0.2 | Stable |
| Non-ICU | Linear | 1 | 1080.2 | -5.7 | -8.4 to -3.0 | Decreasing |
| Species proportions | | | | | | |
| Enterobacterales | | | | | | |
| Enterobacter cloacae | Linear | 1 | 721.4 | -4.6 | -10.8 to 1.7 | Stable |
| Escherichia coli | 2020 | 2 | 1776.8 | 2.6 | -0.4 to 5.6 | Stable |
| Klebsiella pneumoniae | 2021 | 2 | 2595.3 | 4.8 | 2.3 to 7.3 | Increasing |
| Proteus mirabilis | Linear | 1 | 615.7 | -17.7 | -22.9 to -12.4 | Decreasing |
| Serratia marcescens | 2022 | 2 | 763.2 | 15.8 | 9.1 to 22.5 | Increasing |
| Enterobacterales subtotal | 2015 | 2 | 2361.8 | 0.5 | -0.3 to 1.3 | Stable |
| Non-fermenting GNB | | | | | | |
| Acinetobacter baumannii | Linear | 1 | 1641.3 | -4.0 | -7.0 to -1.1 | Decreasing |
| Pseudomonas aeruginosa | Linear | 1 | 1217.7 | -6.4 | -10.2 to -2.6 | Decreasing |
| Non-fermenting GNB subtotal | 2015 | 2 | 2358.6 | -1.4 | -4.3 to 1.4 | Stable |
| Fluoroquinolone-resistance | 2019, 2021 | 3 | 1873.9 | -3.7 | -5.3 to -2.1 | Decreasing |
| Extended-spectrum cephalosporin-resistance | 2021 | 2 | 1600.2 | -3.5 | -4.9 to -2.0 | Decreasing |
| Carbapenem-resistant isolates | | | | | | |
| All isolates | 2021 | 2 | 2515.2 | 2.5 | 0.5 to 4.6 | Increasing |
| Enterobacterales | 2021 | 2 | 1831.2 | 7.0 | 4.0 to 10.1 | Increasing |
| Acinetobacter baumannii | 2017 | 2 | 261.9 | -0.6 | -2.6 to 1.5 | Stable |
| Klebsiella pneumoniae | 2019 | 2 | 899.9 | 12.4 | 8.2 to 16.6 | Increasing |
| Pseudomonas aeruginosa | Linear | 1 | 245.3 | 5.6 | -0.4 to 11.5 | Stable |
| Difficult-to-treat (all evaluable) | | | | | | |
| All isolates | 2015, 2021 | 3 | 2138.2 | 7.1 | 1.7 to 12.6 | Increasing |
| Enterobacterales | 2016, 2021 | 3 | 1455.9 | 23.8 | 10.9 to 36.8 | Increasing |
| Acinetobacter baumannii | 2020 | 2 | 328.3 | -11.3 | -16.0 to -6.5 | Decreasing |
| Klebsiella pneumoniae | 2021 | 2 | 890.4 | 9.5 | 5.2 to 13.7 | Increasing |
| Abbreviations: AAPC, average annual percentage change; CI, confidence interval; GNB, gram-negative bacilli; ICU, intensive care unit; WBIC, weighted Bayesian Information Criterion. | | | | | | |
| a—Annual percentage change (APC) from segmented binomial regression (log link) with data-driven breakpoints selected by weighted Bayesian Information Criterion (WBIC; Kim et al., 2023); minimum 2 years from study boundaries and 2 years between breakpoints. Grid search over 0-, 1-, and 2-breakpoint configurations; WBIC adapts penalty between Bayesian information criterion (BIC) and BIC₃ (penalty = 3k·ln[n]) based on maximum segment-specific pseudo-R². Average annual percentage change (AAPC) weighted across segments. 95% delta-method confidence intervals (CIs) (Clegg et al., 2009).  b—Trend direction: increasing (CI entirely above 0), decreasing (CI entirely below 0), stable (CI crosses 0). | | | | | | |

**Supplementary Table 7.** Species Proportions and Temporal Trends Among Bloodstream Gram-Negative Bacilli, 2013–2024

| Organism | n (%) | Breakpoint |  | Annual Percentage Change, % (95% CI) ᵃ | | |  | AAPC, % (95% CI) ᵃ | Trend ᵇ |
| --- | --- | --- | --- | --- | --- | --- | --- | --- | --- |
|  |  |  |  | Segment 1 | Segment 2 | Segment 3 |  |  |  |
| Enterobacterales | | | | | | | | | |
| Overall | 1,487 (73.4) | 2015 |  | -10.0 (-14.7 to -5.3) | 3.0 (1.9 to 4.0) | — |  | 0.5 (-0.3 to 1.3) | Stable |
| ICU | 802 (73.0) | Linear |  | 1.1 (-0.1 to 2.4) | — | — |  | 1.1 (-0.1 to 2.4) | Stable |
| Non-ICU | 685 (73.8) | Linear |  | 2.4 (1.2 to 3.6) | — | — |  | 2.4 (1.2 to 3.6) | Increasing |
| Delta (ICU vs Non-ICU) ᶜ | — | — |  | — | — | — |  | -1.3 (-3.0 to 0.5) | Stable |
| Non-fermenting GNB | | | | | | | | | |
| Overall | 540 (26.6) | 2015 |  | 35.9 (13.5 to 58.3) | -8.2 (-10.8 to -5.6) | — |  | -1.4 (-4.3 to 1.4) | Stable |
| ICU | 297 (27.0) | Linear |  | -2.7 (-5.6 to 0.2) | — | — |  | -2.7 (-5.6 to 0.2) | Stable |
| Non-ICU | 243 (26.2) | Linear |  | -5.7 (-8.4 to -3.0) | — | — |  | -5.7 (-8.4 to -3.0) | Decreasing |
| Delta (ICU vs Non-ICU) ᶜ | — | — |  | — | — | — |  | 3.0 (-0.9 to 6.9) | Stable |
| Species Distribution | | | | | | | | | |
| Enterobacterales | | | | | | | | | |
| Enterobacter cloacae | 83 (4.1) | Linear |  | -4.6 (-10.8 to 1.7) | — | — |  | -4.6 (-10.8 to 1.7) | Stable |
| Escherichia coli | 315 (15.5) | 2020 |  | -7.2 (-12.3 to -2.1) | 22.3 (11.6 to 32.9) | — |  | 2.6 (-0.4 to 5.6) | Stable |
| Klebsiella pneumoniae | 763 (37.6) | 2021 |  | 16.7 (13.2 to 20.2) | -21.4 (-27.1 to -15.7) | — |  | 4.8 (2.3 to 7.3) | Increasing |
| Proteus mirabilis | 71 (3.5) | Linear |  | -17.7 (-22.9 to -12.4) | — | — |  | -17.7 (-22.9 to -12.4) | Decreasing |
| Serratia marcescens | 94 (4.6) | 2022 |  | -1.6 (-10.1 to 7.0) | 140.2 (61.1 to 219.3) | — |  | 15.8 (9.1 to 22.5) | Increasing |
| Subtotal | 1,487 (73.4) | 2015 |  | -10.0 (-14.7 to -5.3) | 3.0 (1.9 to 4.0) | — |  | 0.5 (-0.3 to 1.3) | Stable |
| Non-fermenting GNB | | | | | | | | | |
| Acinetobacter baumannii | 277 (13.7) | Linear |  | -4.0 (-7.0 to -1.1) | — | — |  | -4.0 (-7.0 to -1.1) | Decreasing |
| Pseudomonas aeruginosa | 176 (8.7) | Linear |  | -6.4 (-10.2 to -2.6) | — | — |  | -6.4 (-10.2 to -2.6) | Decreasing |
| Subtotal | 540 (26.6) | 2015 |  | 35.9 (13.5 to 58.3) | -8.2 (-10.8 to -5.6) | — |  | -1.4 (-4.3 to 1.4) | Stable |
| Abbreviations: AAPC, average annual percentage change; CI, confidence interval; GNB, gram-negative bacilli; ICU, intensive care unit. | | | | | | | | | |
| a—Annual percentage change (APC) from segmented binomial regression (log link) with data-driven breakpoints selected by weighted Bayesian Information Criterion (WBIC; Kim et al., 2023); minimum 2 years from study boundaries and 2 years between breakpoints. Average annual percentage change (AAPC) weighted across segments. 95% delta-method confidence intervals (CIs) (Clegg et al., 2009).  b—Trend direction: increasing (CI entirely above 0), decreasing (CI entirely below 0), stable (CI crosses 0).  c—Delta: difference in AAPC between intensive care unit (ICU) and non-ICU settings (ICU minus non-ICU). | | | | | | | | | |

**Supplementary Figure 2.** Species-Specific Annual Proportions Among Bloodstream Gram-Negative Bacilli. Heatmap displaying the annual proportion (%) of each species relative to all blood gram-negative bacilli isolates (N = 2,027). Abbreviations: GNB, gram-negative bacilli; NFGNB, non-fermenting gram-negative bacilli. Species: A. baumannii, Acinetobacter baumannii; E. cloacae, Enterobacter cloacae; E. coli, Escherichia coli; K. aerogenes, Klebsiella aerogenes; K. oxytoca , Klebsiella oxytoca; K. pneumoniae, Klebsiella pneumoniae; M. morganii, Morganella morganii; P. aeruginosa, Pseudomonas aeruginosa; P. mirabilis, Proteus mirabilis; P. stuartii, Providencia stuartii; S. marcescens, Serratia marcescens.

**Supplementary Figure 3.** Temporal Trends of Species-Level Proportions Among Bloodstream Gram-Negative Bacilli. Observed proportions (connected markers) with segmented binomial regression trend lines and 95% confidence bands. Average annual percentage change (AAPC) with 95% confidence intervals is annotated in each panel. Vertical dashed lines indicate data-driven breakpoints.

**Supplementary Figure 4.** Temporal Trends of Resistance Phenotype Proportions Among Bloodstream Gram-Negative Bacilli. Observed proportions (connected markers) with segmented binomial regression trend lines and 95% confidence bands. Average annual percentage change (AAPC) with 95% confidence intervals is annotated in each panel. Vertical dashed lines indicate data-driven breakpoints. Abbreviations: CRAB, carbapenem-resistant Acinetobacter baumannii; CRE, carbapenem-resistant Enterobacterales; CRPA, carbapenem-Resistant Pseudomonas aeruginosa; DTR, difficult-to-treat resistance.

**Supplementary Table 8.** Linearity Testing for Continuous Variables in 30-Day Mortality Models Among Bloodstream Gram-Negative Bacilli, 2013–2024 ᵃ

| Variable | Linear AIC | Alternative 1 AIC ᵇ | Alternative 2 AIC ᵇ | ΔAIC | P (non-linearity) | Chosen Form ᶜ |
| --- | --- | --- | --- | --- | --- | --- |
| Age (per SD) | 2,082.2 | 2,080.7 | 2,081.9 | 1.5 | 0.011 | RCS(3) |
| Elixhauser comorbidity index (van Walraven) ᵈ | 2,081.9 | 2,081.8 | 2,083.5 | 0.1 | — | Linear |
| Year (calendar) | 2,082.2 | 2,080.6 | 2,082.4 | 1.6 | 0.011 | RCS(3) |
| Abbreviations: AIC, Akaike Information Criterion; ΔAIC, change in Akaike Information Criterion; RCS, restricted cubic spline; SD, standard deviation. | | | | | | |
| a—All models adjusted for: organism-resistance composite, age, sex, intensive care unit (ICU) at culture, BSI source identified, healthcare-associated infection, COVID-19 era, Elixhauser Comorbidity Index (ECI; van Walraven weighting), and calendar year.  b—For age and year: Alternative 1 = restricted cubic spline (RCS; 3 knots), Alternative 2 = RCS (4 knots). For Elixhauser Comorbidity Index (ECI): Alternative 1 = fractional polynomial (FP) degree 1, Alternative 2 = FP degree 2.  c—Selection rule: non-linear form preferred if change in Akaike Information Criterion (ΔAIC) > 2 (linear minus best alternative) or Wald P < 0.05 for non-linearity; otherwise linear retained (parsimony).  d—Elixhauser Comorbidity Index (ECI) tested via fractional polynomial (FP) instead of restricted cubic spline (RCS) due to zero-inflated distribution; FP handles concentrated data more robustly. Raw ECI scale used for FP testing; Winsorized standardized (per SD) form used when linear term selected. | | | | | | |

**Supplementary Figure 5.** Restricted Cubic Spline Dose-Response Curves for Continuous Covariables and 30-Day Mortality Among Bloodstream Gram-Negative Bacilli. (A) Age (winsorized standardized, SD units) with reference at 0 (population mean). (B) Calendar year with reference at 2018. Curves are derived from the primary composite model (modified Poisson regression with robust standard errors clustered by patient) using 3-knot restricted cubic splines. Solid lines represent adjusted risk ratios; shaded bands represent 95% confidence intervals estimated from the variance-covariance matrix. Dots indicate knot positions. Vertical dashed lines mark the reference value. Abbreviations: SD, standard deviation.

**Supplementary Table 9.** Multivariable Predictors of 30-Day Mortality Among Bloodstream Gram-Negative Bacilli, 2013–2024 ᵃ

| Variable | N | Died n (%) | Crude RR (95% CI) | aRR (95% CI) | P value |
| --- | --- | --- | --- | --- | --- |
| Organism-Resistance Composite | | | | | |
| Non-difficult-to-treat | | | | | |
| Enterobacterales | | | | | |
| Carbapenem-susceptible (ref.) | 727 | 192 (26.4) | 1.00 | 1.00 | — |
| Carbapenem-resistant | 127 | 58 (45.7) | 1.73 (1.38 to 2.17) | 1.28 (1.03 to 1.60) | 0.028 |
| Non-fermenting GNB | | | | | |
| Carbapenem-susceptible | 136 | 32 (23.5) | 0.89 (0.64 to 1.23) | 0.92 (0.68 to 1.24) | 0.577 |
| Carbapenem-resistant | 67 | 21 (31.3) | 1.19 (0.82 to 1.73) | 0.91 (0.64 to 1.29) | 0.593 |
| Difficult-to-treat | | | | | |
| Enterobacterales | 334 | 189 (56.6) | 2.14 (1.84 to 2.50) | 1.35 (1.15 to 1.59) | <0.001 |
| Non-fermenting GNB | 182 | 89 (48.9) | 1.85 (1.53 to 2.24) | 1.31 (1.09 to 1.57) | 0.005 |
| Demographics | | | | | |
| Age ᵇ | 1573 | — | 1.31 (1.23 to 1.41) | Non-linear ᵇ | <0.001 |
| Women | 537 | 201 (37.4) | 1.02 (0.89 to 1.17) | 1.13 (1.00 to 1.27) | 0.051 |
| Clinical Setting | | | | | |
| Intensive care unit at culture | 805 | 465 (57.8) | 3.82 (3.20 to 4.57) | 3.20 (2.63 to 3.89) | <0.001 |
| BSI source identified | 677 | 283 (41.8) | 1.26 (1.10 to 1.43) | 0.92 (0.82 to 1.03) | 0.148 |
| Healthcare-associated | 1076 | 475 (44.1) | 2.07 (1.73 to 2.48) | 1.18 (0.98 to 1.42) | 0.088 |
| Temporal | | | | | |
| COVID-19 era (2020–2024) | 860 | 343 (39.9) | 1.19 (1.05 to 1.36) | 1.12 (0.91 to 1.38) | 0.275 |
| Year ᵇ | 1573 | — | 1.01 (0.99 to 1.03) | Non-linear ᵇ | 0.053 |
| Comorbidity | | | | | |
| Elixhauser Comorbidity Index (per SD) | 1573 | — | 1.03 (0.97 to 1.09) | 1.07 (1.01 to 1.12) | 0.019 |
| Abbreviations: aRR, adjusted risk ratio; CI, confidence interval; GNB, gram-negative bacilli; RCS, restricted cubic spline; RR, risk ratio; SD, standard deviation. | | | | | |
| a—30-day in-hospital mortality; restricted to first culture per admission (N = 1,573); modified Poisson regression with robust standard errors clustered by patient. Age and Elixhauser Comorbidity Index (ECI) entered as Winsorized standardized variables (per standard deviation); non-linear forms applied where indicated by linearity testing (Supplementary Table 8). Model Akaike Information Criterion (AIC) = 2,079.8.  b—Variables modeled with restricted cubic splines where non-linearity was detected; linearity testing results reported in Supplementary Table 8. Risk ratio (RR) not shown for non-linear terms; P value represents the Wald test for overall variable effect. | | | | | |

**Supplementary Figure 6.** Forest Plot of Adjusted Risk Ratios for 30-Day Mortality by Composite Resistance–Organism Classification Among Bloodstream Gram-Negative Bacilli. Adjusted risk ratios with 95% confidence intervals (N = 1,573 first bloodstream cultures per admission). The primary exposure is a six-level composite variable crossing organism group (Enterobacterales vs non-fermenting gram-negative bacilli) with resistance hierarchy (carbapenem-susceptible, carbapenem-resistant but not difficult-to-treat, and difficult-to-treat). Reference category: carbapenem-susceptible Enterobacterales. All models adjusted for age, sex, intensive care unit admission at culture, identified BSI source, healthcare-associated infection, COVID-19 era, year, and Elixhauser Comorbidity Index. Abbreviations: aRR, adjusted risk ratio; BSI, bloodstream infection; CI, confidence interval; DTR, difficult-to-treat resistance; GNB, gram-negative bacilli; ICU, intensive care unit.

**Supplementary Table 10.** Resistance Hierarchy and 30-Day Mortality Among Bloodstream Gram-Negative Bacilli, 2013–2024 ᵃ

| Variable | N | Died n (%) | Crude RR (95% CI) | aRR (95% CI) | P value |
| --- | --- | --- | --- | --- | --- |
| Resistance Hierarchy ᵇ | | | | | |
| Non-resistant (ref.) | 460 | 103 (22.4) | 1.00 | 1.00 | — |
| Fluoroquinolone-resistance | 162 | 51 (31.5) | 1.41 (1.06 to 1.87) | 1.29 (1.00 to 1.67) | 0.050 |
| Extended-spectrum cephalosporin-resistance ᶜ | 293 | 83 (28.3) | 1.27 (0.99 to 1.62) | 1.15 (0.90 to 1.46) | 0.264 |
| Carbapenem-resistance | 232 | 92 (39.7) | 1.77 (1.40 to 2.23) | 1.31 (1.04 to 1.64) | 0.019 |
| Difficult-to-treat | 516 | 278 (53.9) | 2.41 (1.99 to 2.91) | 1.53 (1.27 to 1.84) | <0.001 |
| Organism Group | | | | | |
| Non-fermenting GNB (vs Enterobacterales) | 430 | 153 (35.6) | 0.97 (0.83 to 1.12) | 0.92 (0.80 to 1.05) | 0.194 |
| Demographics | | | | | |
| Age ᵈ | 1663 | — | 1.31 (1.22 to 1.40) | Non-linear ᵈ | <0.001 |
| Women | 571 | 211 (37.0) | 1.02 (0.89 to 1.16) | 1.13 (1.00 to 1.27) | 0.047 |
| Clinical Setting | | | | | |
| Intensive care unit at culture | 846 | 486 (57.4) | 3.88 (3.26 to 4.62) | 3.28 (2.71 to 3.97) | <0.001 |
| BSI source identified | 699 | 288 (41.2) | 1.25 (1.10 to 1.41) | 0.91 (0.81 to 1.02) | 0.102 |
| Healthcare-associated | 1140 | 495 (43.4) | 2.03 (1.70 to 2.42) | 1.14 (0.95 to 1.37) | 0.147 |
| Temporal | | | | | |
| COVID-19 era (2020–2024) | 901 | 356 (39.5) | 1.20 (1.05 to 1.37) | 1.13 (0.92 to 1.39) | 0.239 |
| Year ᵈ | 1663 | — | 1.01 (0.99 to 1.03) | Non-linear ᵈ | 0.058 |
| Comorbidity | | | | | |
| Elixhauser Comorbidity Index (per SD) | 1663 | — | 1.04 (0.98 to 1.10) | 1.07 (1.02 to 1.13) | 0.011 |
| Abbreviations: aRR, adjusted risk ratio; CI, confidence interval; GNB, gram-negative bacilli; ICU, intensive care unit; RR, risk ratio; SD, standard deviation. | | | | | |
| a—30-day in-hospital mortality; restricted to first culture per admission (N = 1,663); modified Poisson regression with robust standard errors clustered by patient. Age and Elixhauser Comorbidity Index (ECI) entered as Winsorized standardized variables (per standard deviation); non-linear forms applied where indicated by linearity testing (Supplementary Table 8). Model Akaike Information Criterion (AIC) = 2,182.1.  b—Resistance classified using a 5-level mutually exclusive hierarchy: non-resistant, fluoroquinolone-resistant (FQR), extended-spectrum cephalosporin-resistant (ESC-R), carbapenem-resistant, and difficult-to-treat resistance (DTR) (Kadri et al., 2018).  c—ESC-R classification applies to Enterobacterales only; non-fermenting gram-negative bacilli (GNB) classified as non-resistant, FQR, carbapenem-resistant, or DTR.  d—Variables modeled with restricted cubic splines (3 knots) where non-linearity was detected; linearity testing results reported in Supplementary Table 8. Risk ratio (RR) not shown for non-linear terms; P value represents the Wald test for overall variable effect. | | | | | |

**Supplementary Table 11.** First-Line Active Agents and 30-Day Mortality Among Bloodstream Gram-Negative Bacilli, 2013–2024 ᵃ ᵇ

| Variable | N | Died n (%) | Crude RR (95% CI) | aRR (95% CI) | P value |
| --- | --- | --- | --- | --- | --- |
| First-Line Active Categories ᶜ ᵈ | | | | | |
| Adequate (3–5 active; ref.) | 580 | 137 (23.6) | 1.00 | 1.00 | — |
| Limited (1–2 active) | 347 | 127 (36.6) | 1.55 (1.27 to 1.90) | 1.24 (1.02 to 1.51) | 0.033 |
| Difficult-to-treat (0 active) | 516 | 278 (53.9) | 2.28 (1.93 to 2.70) | 1.46 (1.23 to 1.73) | <0.001 |
| Organism Group | | | | | |
| Non-fermenting GNB (vs Enterobacterales) | 383 | 141 (36.8) | 0.97 (0.84 to 1.13) | 0.94 (0.83 to 1.08) | 0.399 |
| Demographics | | | | | |
| Age ᵉ | 1443 | — | 1.33 (1.24 to 1.42) | Non-linear ᵉ | <0.001 |
| Women | 482 | 183 (38.0) | 1.02 (0.88 to 1.17) | 1.09 (0.96 to 1.23) | 0.179 |
| Clinical Setting | | | | | |
| Intensive care unit at culture | 762 | 438 (57.5) | 3.76 (3.12 to 4.54) | 3.06 (2.49 to 3.76) | <0.001 |
| BSI source identified | 623 | 271 (43.5) | 1.32 (1.15 to 1.50) | 0.95 (0.84 to 1.06) | 0.352 |
| Healthcare-associated | 1015 | 450 (44.3) | 2.06 (1.70 to 2.50) | 1.17 (0.96 to 1.42) | 0.122 |
| Temporal | | | | | |
| COVID-19 era (2020–2024) | 783 | 324 (41.4) | 1.25 (1.09 to 1.44) | 1.15 (0.92 to 1.43) | 0.218 |
| Year ᵉ | 1443 | — | 1.01 (0.99 to 1.03) | Non-linear ᵉ | 0.086 |
| Comorbidity | | | | | |
| Elixhauser Comorbidity Index (per SD) | 1443 | — | 1.03 (0.96 to 1.09) | 1.06 (1.00 to 1.12) | 0.037 |
| Abbreviations: aRR, adjusted risk ratio; CI, confidence interval; COVID, coronavirus disease 2019; GNB, gram-negative bacilli; RR, risk ratio; SD, standard deviation. | | | | | |
| a—30-day in-hospital mortality; restricted to first culture per admission (N = 1,443); modified Poisson regression with robust standard errors clustered by patient. Age and Elixhauser Comorbidity Index (ECI) entered as Winsorized standardized variables (per standard deviation); non-linear forms applied where indicated by linearity testing (Supplementary Table 8). Model Akaike Information Criterion (AIC) = 1,925.2.  b—N = 1,443 restricted to isolates with susceptibility data for ≥3 mandatory first-line categories (carbapenems, extended-spectrum cephalosporins, fluoroquinolones); piperacillin-tazobactam and the 5th agent counted when reported. 285 admissions excluded due to incomplete susceptibility data.  c—Five first-line antibiotic categories were evaluated for activity: (1) carbapenems (imipenem, meropenem, ertapenem), (2) extended-spectrum cephalosporins (ceftriaxone, cefotaxime, ceftazidime, cefepime), (3) fluoroquinolones (ciprofloxacin, levofloxacin), (4) piperacillin-tazobactam (when tested), and (5) aztreonam for non-Acinetobacter species or ampicillin-sulbactam for Acinetobacter baumannii (when tested). Analysis restricted to Enterobacterales, Pseudomonas aeruginosa, and Acinetobacter baumannii (reference group: ≥3 active categories; Kadri et al., 2018).  d—P-for-trend < 0.001 (ordinal contrast across active-category groups).  e—Variables modeled with restricted cubic splines (3 knots) where non-linearity was detected; linearity testing results reported in Supplementary Table 8. Risk ratio (RR) not shown for non-linear terms; P value represents the Wald test for overall variable effect. | | | | | |

**Supplementary Figure 7.** Dose-Response Relationship Between First-Line Active Antimicrobial Categories and 30-Day Mortality Among Bloodstream Gram-Negative Bacilli. Adjusted risk ratios with 95% confidence intervals for 30-day mortality across three levels of first-line active agent availability: adequate (3–5 active categories; reference), limited (1–2 active), and difficult-to-treat resistance (0 active). First-line categories counted are carbapenems, extended-spectrum cephalosporins, fluoroquinolones, piperacillin-tazobactam, and aztreonam (or ampicillin-sulbactam for Acinetobacter baumannii). Modified Poisson regression adjusted for organism group, age, sex, intensive care unit admission at culture, identified BSI source, healthcare-associated infection, COVID-19 era, Elixhauser Comorbidity Index, and year. Abbreviations: aRR, adjusted risk ratio; BSI, bloodstream infection; CI, confidence interval; DTR, difficult-to-treat resistance.

**Supplementary Table 12.** Sensitivity and Alternative Model Analyses for 30-Day Mortality Among Bloodstream Gram-Negative Bacilli, 2013–2024 ᵃ

| Part A: Composite Exposure Across Sensitivity Specifications, aRR (95% CI) | | | | | | |
| --- | --- | --- | --- | --- | --- | --- |
| Variable | A: Primary | B: In-hospital | C: First admission | D: No ICU | E: No BSI source | F: Last culture |
| Non-difficult-to-treat | | | | | | |
| Carbapenem-resistant Enterobacterales | 1.28 (1.03 to 1.60) | 1.18 (0.98 to 1.43) | 1.29 (1.03 to 1.62) | 1.43 (1.13 to 1.80) | 1.26 (1.01 to 1.57) | 1.13 (0.91 to 1.40) |
| Carbapenem-susceptible non-fermenting GNB | 0.92 (0.68 to 1.24) | 0.90 (0.70 to 1.16) | 0.93 (0.69 to 1.26) | 0.86 (0.63 to 1.18) | 0.93 (0.69 to 1.25) | 0.88 (0.67 to 1.17) |
| Carbapenem-resistant non-fermenting GNB | 0.91 (0.64 to 1.29) | 0.85 (0.63 to 1.15) | 0.94 (0.66 to 1.33) | 1.01 (0.70 to 1.45) | 0.91 (0.64 to 1.29) | 0.82 (0.58 to 1.16) |
| Difficult-to-treat | | | | | | |
| Enterobacterales | 1.35 (1.15 to 1.59) | 1.18 (1.03 to 1.36) | 1.36 (1.16 to 1.59) | 1.63 (1.37 to 1.93) | 1.33 (1.13 to 1.55) | 1.20 (1.04 to 1.40) |
| Non-fermenting GNB | 1.31 (1.09 to 1.57) | 1.17 (1.00 to 1.37) | 1.31 (1.09 to 1.58) | 1.54 (1.27 to 1.86) | 1.29 (1.08 to 1.56) | 1.28 (1.08 to 1.53) |
| Model G (resistance hierarchy × organism group interaction): Wald test P = 0.112. Interaction terms not tabulated; organism-stratified estimates in the exported coefficient dataset. | | | | | | |
| Part B: Staged Regression Decomposition (Composite Model) ᵇ | | | | | | |
| Variable | S1: Base | S2: +ICU | S3: +BSI source (full) |  |  |  |
| Difficult-to-treat | | | |  |  |  |
| Enterobacterales | 1.61 (1.36 to 1.91) | 1.33 (1.13 to 1.55) | 1.35 (1.15 to 1.59) |  |  |  |
| Non-fermenting GNB | 1.53 (1.27 to 1.86) | 1.29 (1.08 to 1.56) | 1.31 (1.09 to 1.57) |  |  |  |
| Abbreviations: aRR, adjusted risk ratio; BSI, bloodstream infection; CI, confidence interval; GNB, gram-negative bacilli; ICU, intensive care unit. | | | | | | |
| a—All models: modified Poisson regression with exponentiated form (adjusted risk ratio [aRR]). Reference group: carbapenem-susceptible Enterobacterales. Clustered by patient (except Model C: robust standard errors, 1 observation/patient).  b—S1 = age, sex, healthcare-associated infection, COVID-19 era, calendar year, Elixhauser Comorbidity Index (ECI). S2 = S1 + intensive care unit (ICU) at culture. S3 = S2 + BSI source identified (full model). | | | | | | |

**Supplementary Figure 8.** Staged Regression Decomposition of Difficult-to-Treat Resistance and 30-Day Mortality Among Bloodstream Gram-Negative Bacilli. Connected dot plots showing the attenuation of adjusted risk ratios across three nested models for the two difficult-to treat resistance (DTR) categories: (A) DTR Enterobacterales and (B) DTR non-fermenting gram-negative bacilli. All models use modified Poisson regression with robust standard errors clustered by patient. Stage 1 (Base) adjusts for age, sex, healthcare-associated infection, COVID-19 era, year, and Elixhauser Comorbidity Index. Stage 2 (+ICU) adds intensive care unit admission at culture. Stage 3 (+BSI) adds identified BSI source (full model). Percentage annotations between stages indicate the proportional change in the risk ratio point estimate. Reference category: carbapenem-susceptible Enterobacterales. Abbreviations: BSI, bloodstream infection; CI, confidence interval; DTR, difficult-to-treat resistance.

**Supplementary Figure 9.** Sensitivity Analysis Comparison of Difficult-to-Treat Resistance and 30-Day Mortality Across Model Specifications Among Bloodstream Gram-Negative Bacilli. Forest plot comparing adjusted risk ratios for difficult-to-treat Enterobacterales and difficult-to treat non-fermenting gram-negative bacilli across six model specifications: primary model (30-day mortality), in-hospital mortality as outcome, first admission only (one observation per patient, robust standard errors), intensive care unit covariable removed, identified BSI source covariable removed, and last culture per admission instead of first. All models use modified Poisson regression with the six-level composite exposure variable. Reference category: carbapenem-susceptible Enterobacterales. Abbreviations: aRR, adjusted risk ratio; BSI, bloodstream infection; CI, confidence interval; DTR, difficult-to-treat resistance; GNB, gram-negative bacilli; ICU, intensive care unit.

**Supplementary Table 13.** Subgroup Trend Analyses (AAPC) by Sex and ICU Status Among Bloodstream Gram-Negative Bacilli, 2013–2024

| Category | Sex (AAPC% [95% CI]) | | |  | Intensive care unit (AAPC% [95% CI]) | | |
| --- | --- | --- | --- | --- | --- | --- | --- |
|  | Men | Women | ΔAAPC (Men – Women; % [95% CI]) |  | ICU | Non-ICU | ΔAAPC (ICU – Non-ICU; % [95% CI]) |
| Organism proportions | | | | | | | |
| Enterobacterales | 2.1 (0.9 to 3.2) | 1.3 (-0.0 to 2.7) | 0.5 (-0.2 to 1.2) |  | 1.1 (-0.1 to 2.4) | 2.4 (1.2 to 3.6) | -0.1 (-1.1 to 0.8) |
| Escherichia coli | 3.6 (-1.5 to 8.7) | 0.3 (-5.2 to 5.8) | 1.1 (-1.2 to 3.3) |  | -4.2 (-9.5 to 1.1) | 6.8 (3.0 to 10.5) | -4.8 (-7.8 to -1.8) |
| Klebsiella pneumoniae | 5.9 (2.8 to 9.0) | 2.8 (-1.4 to 6.9) | 0.1 (-3.3 to 3.6) |  | 6.8 (3.6 to 10.0) | 2.6 (-1.2 to 6.4) | 2.6 (-1.4 to 6.7) |
| Proteus mirabilis | -21.1 (-27.4 to -14.8) | -12.2 (-21.0 to -3.5) | 7.5 (1.8 to 13.2) |  | -16.2 (-23.4 to -9.1) | -19.2 (-27.1 to -11.3) | -0.9 (-6.4 to 4.6) |
| Non-fermenting GNB | -2.7 (-5.9 to 0.5) | -3.5 (-6.9 to -0.1) | 0.4 (-2.8 to 3.7) |  | -2.7 (-5.6 to 0.2) | -5.7 (-8.4 to -3.0) | 1.7 (-1.6 to 5.1) |
| Acinetobacter baumannii | -4.7 (-8.1 to -1.2) | -2.7 (-8.3 to 2.8) | -3.2 (-8.9 to 2.6) |  | -3.2 (-7.1 to 0.8) | -5.5 (-10.1 to -0.9) | 1.0 (-4.1 to 6.2) |
| Pseudomonas aeruginosa | -6.2 (-11.2 to -1.3) | -6.5 (-12.4 to -0.7) | 2.6 (-1.5 to 6.7) |  | -6.0 (-11.8 to -0.3) | -6.5 (-11.6 to -1.5) | 1.1 (-3.5 to 5.6) |
| Resistance phenotypes | | | | | | | |
| Fluoroquinolone resistance | -1.9 (-3.9 to 0.0) | -4.6 (-7.5 to -1.7) | 0.6 (-0.7 to 1.9) |  | -2.7 (-4.4 to -1.0) | -2.0 (-3.8 to -0.2) | -1.0 (-2.3 to 0.4) |
| Extended-spectrum cephalosporin resistance | -2.9 (-4.7 to -1.2) | -6.1 (-9.3 to -2.9) | 1.8 (0.5 to 3.1) |  | 0.0 (-1.2 to 1.2) | -3.0 (-5.0 to -0.9) | 2.4 (1.0 to 3.8) |
| Carbapenem-resistance | | | | | | | |
| Acinetobacter baumannii | -1.2 (-2.7 to 0.3) | -0.7 (-2.9 to 1.5) | 0.9 (-3.2 to 5.1) |  | 4.6 (-0.9 to 10.2) | -4.0 (-6.6 to -1.3) | 2.0 (-1.0 to 5.0) |
| Klebsiella pneumoniae | 12.5 (7.3 to 17.7) | 9.3 (1.4 to 17.2) | 4.2 (-2.6 to 11.1) |  | 12.4 (7.9 to 16.9) | 4.4 (0.0 to 8.7) | 3.9 (-2.1 to 10.0) |
| Pseudomonas aeruginosa | 5.9 (-1.7 to 13.5) | 5.4 (-4.4 to 15.2) | -0.4 (-6.7 to 5.9) |  | 3.7 (-3.2 to 10.6) | 7.8 (-2.9 to 18.5) | 9.6 (1.9 to 17.4) |
| Enterobacterales | 12.5 (8.3 to 16.7) | 3.8 (-1.8 to 9.3) | 3.5 (-1.1 to 8.0) |  | 14.0 (9.7 to 18.3) | 4.7 (0.7 to 8.6) | 2.1 (-2.3 to 6.4) |
| Overall | 3.2 (0.6 to 5.7) | 1.3 (-2.4 to 4.9) | -0.8 (-4.2 to 2.5) |  | 7.9 (5.4 to 10.3) | 0.7 (-2.0 to 3.5) | 2.8 (-0.2 to 5.8) |
| Difficult-to-treat resistance | | | | | | | |
| Acinetobacter baumannii | -11.4 (-17.0 to -5.9) | -5.4 (-9.4 to -1.3) | 4.9 (-0.1 to 9.9) |  | -8.2 (-13.5 to -2.9) | -15.8 (-23.8 to -7.7) | 4.8 (0.5 to 9.2) |
| Klebsiella pneumoniae | 61.6 (6.2 to 117.0) | 11.9 (3.8 to 19.9) | 27.3 (8.7 to 45.9) |  | 15.3 (9.4 to 21.2) | 6.4 (0.5 to 12.4) | -21.4 (-45.8 to 3.1) |
| Pseudomonas aeruginosa | 3.6 (-11.9 to 19.2) | -8.2 (-17.2 to 0.8) | -0.2 (-11.1 to 10.7) |  | -4.1 (-15.6 to 7.3) | -0.8 (-14.9 to 13.2) | 0.5 (-11.9 to 12.9) |
| Enterobacterales | 28.4 (8.2 to 48.7) | 7.6 (-0.1 to 15.4) | 10.8 (-4.2 to 25.7) |  | 11.1 (6.0 to 16.2) | 42.8 (6.9 to 78.6) | -3.4 (-12.1 to 5.4) |
| Overall | 3.6 (0.3 to 6.9) | -0.7 (-5.9 to 4.5) | 3.0 (-3.1 to 9.1) |  | 6.6 (3.4 to 9.8) | 6.5 (-0.2 to 13.2) | 4.1 (-1.0 to 9.1) |
| Abbreviations: AAPC, average annual percentage change; CI, confidence interval; GNB, gram-negative bacilli; ICU, intensive care unit; ΔAAPC, difference in AAPC between subgroups. | | | | | | | |

**Supplementary Table 14.** Trend Analysis Feasibility Assessment Among Bloodstream Gram-Negative Bacilli, 2013–2024

| Outcome | Total Nᵃ | Events (%)ᵇ |  | Sex | | Intensive care unit | |  | Trend analysisᶜ | | |
| --- | --- | --- | --- | --- | --- | --- | --- | --- | --- | --- | --- |
|  |  |  |  | Men | Women | ICU | Non-ICU |  | Overallᵈ | Sexᵉ | Settingᵉ |
| Organism proportions | | | | | | | | | | | |
| Enterobacterales | 2,027 | 1487 (73.4%) |  | 964 | 523 | 802 | 685 |  | Included | Included | Included |
| Non-fermenting GNB | 2,027 | 540 (26.6%) |  | 361 | 179 | 297 | 243 |  | Included | Included | Included |
| Species proportions | | | | | | | | | | | |
| Enterobacterales | | | | | | | | | | | |
| Enterobacter cloacae | 2,027 | 83 (4.1%) |  | 53 | 30 | 33 | 50 |  | Included | Excluded (Women < 50) | Excluded (ICU < 50) |
| Escherichia coli | 2,027 | 315 (15.5%) |  | 178 | 137 | 92 | 223 |  | Included | Included | Included |
| Klebsiella aerogenes | 2,027 | 34 (1.7%) |  | 26 | 8 | 17 | 17 |  | Excluded (n = 34) | Excluded (Men < 50, Women < 50) | Excluded (ICU < 50, Non-ICU < 50) |
| Klebsiella oxytoca | 2,027 | 13 (0.6%) |  | 8 | 5 | 6 | 7 |  | Excluded (n = 13) | Excluded (Men < 50, Women < 50) | Excluded (ICU < 50, Non-ICU < 50) |
| Klebsiella pneumoniae | 2,027 | 763 (37.6%) |  | 506 | 257 | 507 | 256 |  | Included | Included | Included |
| Morganella morganii | 2,027 | 15 (0.7%) |  | 11 | 4 | 12 | 3 |  | Excluded (n = 15) | Excluded (Men < 50, Women < 50) | Excluded (ICU < 50, Non-ICU < 50) |
| Proteus mirabilis | 2,027 | 71 (3.5%) |  | 44 | 27 | 37 | 34 |  | Included | Excluded (Men < 50, Women < 50) | Excluded (ICU < 50, Non-ICU < 50) |
| Providencia stuartii | 2,027 | 40 (2.0%) |  | 33 | 7 | 26 | 14 |  | Excluded (n = 40) | Excluded (Men < 50, Women < 50) | Excluded (ICU < 50, Non-ICU < 50) |
| Serratia marcescens | 2,027 | 94 (4.6%) |  | 67 | 27 | 52 | 42 |  | Included | Excluded (Women < 50) | Excluded (Non-ICU < 50) |
| Non-fermenting GNB | | | | | | | | | | | |
| Acinetobacter baumannii | 2,027 | 277 (13.7%) |  | 197 | 80 | 170 | 107 |  | Included | Included | Included |
| Pseudomonas aeruginosa | 2,027 | 176 (8.7%) |  | 112 | 64 | 88 | 88 |  | Included | Included | Included |
| Resistance phenotypes | | | | | | | | | | | |
| Fluoroquinolone resistance | 1,961 | 1228 (62.6%) |  | 814 | 414 | 754 | 474 |  | Included | Included | Included |
| Extended-spectrum cephalosporin resistance | 1,292 | 892 (69.0%) |  | 593 | 299 | 546 | 346 |  | Included | Included | Included |
| Carbapenem-resistant Enterobacterales | 1,447 | 595 (41.1%) |  | 412 | 183 | 427 | 168 |  | Included | Included | Included |
| Carbapenem resistance (overall) | 1,890 | 887 (46.9%) |  | 611 | 276 | 618 | 269 |  | Included | Included | Included |
| Difficult-to-treat resistance (Enterobacterales) | 1,258 | 403 (32.0%) |  | 280 | 123 | 305 | 98 |  | Included | Included | Included |
| Difficult-to-treat resistance (overall) | 1,691 | 608 (36.0%) |  | 425 | 183 | 442 | 166 |  | Included | Included | Included |
| Species-specific resistance | | | | | | | | | | | |
| Carbapenem-resistant isolates | | | | | | | | | | | |
| Acinetobacter baumannii | 272 | 226 (83.1%) |  | 160 | 66 | 151 | 75 |  | Included | Included | Included |
| Escherichia coli | 310 | 18 (5.8%) |  | 11 | 7 | 2 | 16 |  | Excluded (n = 18) | Excluded (Men < 50, Women < 50) | Excluded (ICU < 50, Non-ICU < 50) |
| Klebsiella pneumoniae | 743 | 493 (66.4%) |  | 337 | 156 | 372 | 121 |  | Included | Included | Included |
| Pseudomonas aeruginosa | 171 | 66 (38.6%) |  | 39 | 27 | 40 | 26 |  | Included | Excluded (Men < 50, Women < 50) | Excluded (ICU < 50, Non-ICU < 50) |
| Difficult-to-treat resistance | | | | | | | | | | | |
| Acinetobacter baumannii | 265 | 178 (67.2%) |  | 130 | 48 | 120 | 58 |  | Included | Excluded (Women < 50) | Included |
| Escherichia coli | 225 | 6 (2.7%) |  | 4 | 2 | 1 | 5 |  | Excluded (n = 6) | Excluded (Men < 50, Women < 50) | Excluded (ICU < 50, Non-ICU < 50) |
| Klebsiella pneumoniae | 680 | 368 (54.1%) |  | 252 | 116 | 283 | 85 |  | Included | Included | Included |
| Pseudomonas aeruginosa | 168 | 27 (16.1%) |  | 15 | 12 | 17 | 10 |  | Excluded (n = 27) | Excluded (Men < 50, Women < 50) | Excluded (ICU < 50, Non-ICU < 50) |
| Abbreviations: GNB, gram-negative bacilli; ICU, intensive care unit. | | | | | | | | | | | |
| a—Total N = evaluable isolates (nonmissing outcome).  b—Events = isolates positive for the outcome.  c—Gating criteria applied to all trend analyses (overall, sex, and setting): N ≥ 100 evaluable isolates, prevalence 0.5–99.5%.  d—Included if events ≥ 50.  e—Included if events ≥ 50 in both strata of each comparison (men and women; intensive care unit [ICU] and non-ICU). | | | | | | | | | | | |
